# Systematic Investigation of Cellular Response to Hydroxyl Group Orientation Differences on Gold Glyconanoparticles

**DOI:** 10.1021/acsomega.3c05920

**Published:** 2023-10-31

**Authors:** Melike Sarıçam, Merve Ercan Ayra, Mustafa Culha

**Affiliations:** † Department of Genetics and Bioengineering, Yeditepe University, Istanbul 34755, Turkey; ‡ Department of Chemistry & Biochemistry, Augusta University, Augusta, Georgia 30912, United States; § Sabanci University Nanotechnology Research and Application Center (SUNUM), Istanbul 34956, Turkey

## Abstract

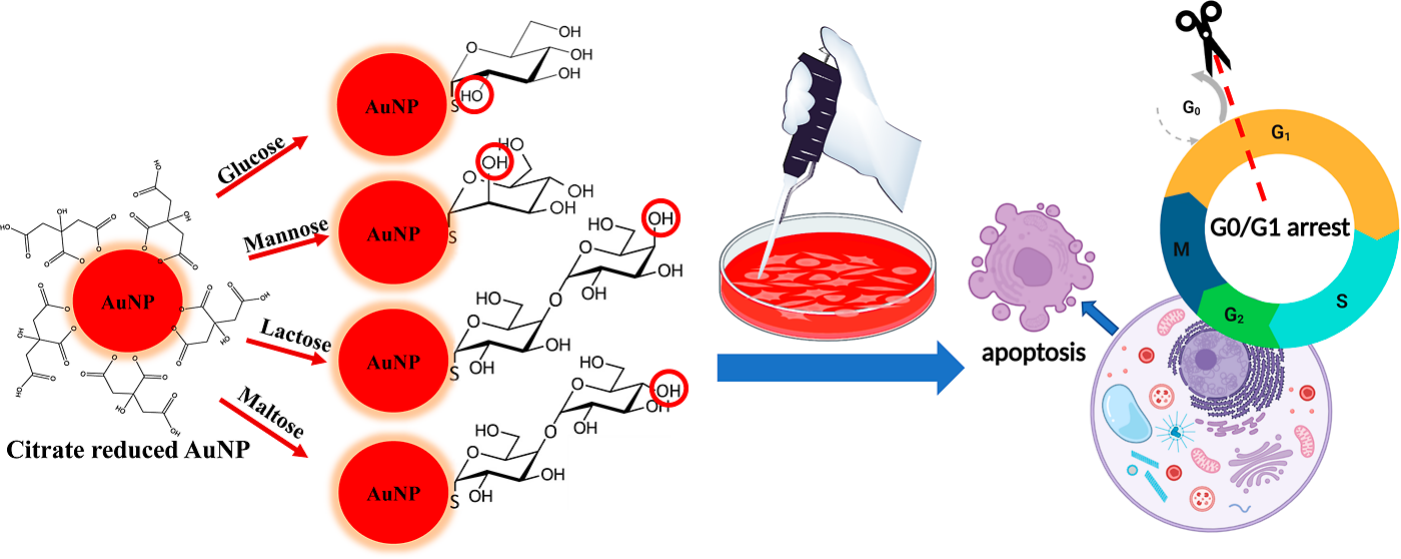

Nanoparticle (NP) surfaces act as the interface as they interact with living systems and play a critical role in defining their cellular response. The nature of these interactions should be well understood to design safer and more effective NPs to be used in a wide range of biomedical applications. At the moment, it is not clear how a subtle change in surface chemistry will affect an NP’s behavior in a biological system. Thus, understanding the role of such a small change is critical and may allow one to fine-tune a biological response. In this study, the cellular response to −OH orientation differences generated on gold glyconanoparticles, which are recently considered promising therapeutic agents as they mimic a glycocalyx, is investigated. As model molecules, glucose and mannose (C2 epimer) as monosaccharides and lactose and maltose (galactose and glucose as free units, C4 epimer) as disaccharides were chosen to monitor the cellular response in A549, BEAS-2b, and MDA-MB-231 cells through cellular uptake, cytotoxicity, and cell cycle progression. The three cell lines gave various and remarkable cellular responses to the same subtle −OH differences on gold glyconanoparticles, and it is determined that not only −OH orientation differences but also the number of saccharides on gold glyconanoparticles affect the cellular response. It was shown that mannose (C2 epimer to glucose) was significant with the promise of being a therapeutic agent for lung cancer therapy, whereas the toxicological profile of MDA-MB-231 cells was affected by AuNPs–glucose the most. This study demonstrates that clearly small chemical alterations on a NP surface can result in a significant cellular response. It can be concluded that the −OH orientation at the second and fourth carbon of a carbohydrate ring has a critical role in designing and engineering novel gold glyconanoparticles (consisting of monolayer mono- or disaccharides) for a specific cancer therapy.

## 1 Introduction

The surface chemistry of nanoparticles (NPs) plays a critical role in creating functionalized nanostructures for safe and effective biomedical applications because the chemical groups or moieties on their surfaces serve as a critical interface defining the nature of their interactions with the surrounding matrix.^1−4^ Over the last couple of decades, research has focused on tailoring the NP surface chemistry to create more functional structures for biomedical applications. These include amino acids,^5^ peptides,^6^ carbohydrates,^7^ lipids,^8^ oligonucleotides,^9^ and antibodies^10^ rather than synthetic materials^11^ due to their distinct biocompatibility. Carbohydrates as the third element of life attract specific attention due to their myriad roles as functional and structural components in biological processes like cell–cell signaling, immune responses, viral and bacterial infections, inflammation, cell proliferation and adhesion, activity of pituitary hormones, and metastasis.^12−14^ Carbohydrate–protein interactions govern all these biological processes with their versatile structure, which includes a hydrogen-bond of networks, glycosidic linkage, and conformation plasticity.^15,16^ Since the binding affinity of carbohydrate–protein interactions is weak (*K*_a_ in the range of ∼10^3^ M^–1^),^17^ carbohydrates are decorated on NPs in order to influence the sensitivity and specificity of binding. For this reason, carbohydrate-conjugated NPs attracted great attention in biomedical applications, such as drug delivery,^18^ biosensors,^19^ molecular therapeutics,^20^ bioimaging,^21^ and vaccine development,^22^ by virtue of their fundamental biological functions.^23^ The term gold glyconanoparticles, which refer to carbohydrate-tailored AuNPs, was brought into existence in 2001 by de la Fuente et al.,^24^ and the number of research studies to investigate their possible potential in biomedical applications gradually increased. The benefits of AuNPs, including easy synthesis in a variety of shapes and sizes, easy surface modification and control over the ligand density on the surface, water dispersibility, higher storage stability, unique optical features, and lack of cytotoxicity, made them one of the most preferred carriers in several studies.^18,25,26^ The main aim to work with gold glyconanoparticles is to mimic the naturally present glycocalyx. Understanding the molecular mechanisms between carbohydrate-decorated AuNPs and their cellular surroundings enables control of the glycocalyx and thus design of novel probes, targeting agents, lectin inhibitors, and drug delivery systems. To date, gold glyconanoparticles in nanomedicine have a notable history.^12,27−30^ In a study, AuNPs having a diameter below 2 nm were modified with lactose, maltose, and glucose neoglycoconjugate as specific tumor-associated carbohydrate antigens and AuNPs–lactose conjugates showed a strong protective effect for lung metastasis in mouse melanoma models (B16F10).^31^ In another study, the utilization of thio-glucose-modified 13 nm AuNPs with megavoltage X-rays enhanced the radiation affect and induced apoptosis in A549 cells.^32^ Furthermore, Suvarna et al. have developed a novel 2-deoxy-d-glucose (2DG)-capped AuNPs as a better candidate for theranostic studies.^33^ In that study, it was claimed that 2DG-AuNPs were a new targeting agent for glucose-dependent cancer cell types and a perfect candidate for AuNPs as a drug to be delivered to the interested sites in comparison to glucose-coated AuNPs. Additionally, for designing a potential carbohydrate-based anticancer vaccine, AuNPs were conjugated with T-cell helper peptides and sialyl-Tn and Lewis antigens, which were tumor-associated carbohydrate antigens overexpressed in several cancer types.^34^ In a recent study, it was claimed that gold glyconanoparticles decorated with condensation products of *N*-acetylamino-d-glucose, d-mannose, d-galactose, and l-fucose with 6-mercaptohexanoic acid hydrazide could be a promising efficient drug for acute respiratory viral infection treatments.^35^ The preliminary data showed that these gold glyconanoparticles at high doses had low toxicity in MDCK cells and also resulted in remarkable antiviral activity against the A/Puerto Rico/8/34 (H1N1) influenza virus at doses of 3 and 6 μg/mL. Last but not least, a glyconanoparticles platform including 31 glycopolymers tailored with heterogeneous or homogeneous different sugar moieties, such as β-glucose, β-galactose, α-mannose, β-*N*-acetyl glucosamine, and β-*N*-acetyl galactosamine (unconjugated with AuNPs), was reported.^36^ The therapeutic and targeting potentials of glyconanoparticles were systematically screened in CT26, DU145, A549, and PC3 tumors, and it was demonstrated that new types of glyconanoparticles had a great potential for the development of cancer-targeting nanomedicines. At this point, it must be mentioned that the common similarity between these studies is the explicit differences in the surface chemistry of AuNPs and the anticipated different cellular responses to them. It is clear that the surface chemistry causing a NP’s surface to become charged or neutral can have a great influence starting from the point where they are added into cell culture media through protein corona formation.^37^ The nature of the protein corona, hard or soft, can have a dramatic influence on the NPs uptake mechanism. On the other hand, a systematic investigation of cellular response to the subtle differences on NPs surface chemistry such as the hydroxyl group orientation of mono- and disaccharides remained unclear.

Here, we report the first systematic screening and therapeutic evaluation of a gold glyconanoparticles-based study focusing on hydroxyl group orientation differences of mono- or disaccharides tailored on the spherical AuNPs surfaces. To achieve this objective, the spherical AuNPs with 13 nm average size were modified with four custom chosen carbohydrates, such as glucose, mannose, lactose, and maltose, and their cellular responses, such as cytotoxicity, cellular uptake, and cell cycle tests, were systematically investigated on A549 (human Caucasian lung carcinoma), BEAS-2b (human bronchial epithelial cell), and MDA-MB-231 (mammalian breast carcinoma) cell lines comparatively. The reason behind why these four carbohydrates were selected was that glucose and mannose were epimers at the second carbon (C2), and the free units of lactose and maltose were galactose and glucose, which were epimers at the fourth carbon (C4). With this, the effect of a small difference, such as in the −OH orientation of glycocalyx-mimicking NPs on either healthy or cancerous cells, can be evaluated. For monolayer coating, mono- and disaccharides were thiolated with Lawesson reagent, which only thiolates C1 of carbohydrates, and thus the cellular response to −OH orientation differences at C2 and C4 on the NP surfaces could be screened. In addition, this study compares the cellular responses to −OH orientation on monosaccharide-coated AuNPs and that of free saccharides on disaccharide-modified AuNPs. When the results were considered, the four gold glyconanoparticles-induced varying cellular responses in three cell lines. AuNPs–mannose (C2 epimer of glucose) and AuNPs–lactose (galactose as the free unit on the surface and C4 epimer of glucose) induced cytotoxicity and influenced the cell cycle progression of A549 cells. However, highly uptaken AuNPs–lactose and AuNPs–maltose conjugates, on which the free units were C4 epimers, caused significant apoptosis and G0/G1 phase cell cycle arrest in BEAS-2b cells. Moreover, glucose- and mannose-functionalized AuNPs, which were C2 epimers, caused a deleterious effect on cellular viability of MDA-MB-231 cells, and also just AuNPs–glucose conjugates arrested MDA-MB-231 cells at the G0/G1 phase. As a result, the cellular response of three cell lines varied according to –OH orientation differences at the second or fourth carbon of these chosen carbohydrates. In light of these results, it is obvious that the –OH orientation at the second and fourth carbons of the carbohydrate had a critical role in designing and engineering novel gold glyconanoparticles (consisting of monolayer mono- or disaccharides) for a specific cancer therapy.

## 2 Methods

### 2.1 AuNPs Synthesis

The spherical AuNPs with a 13 nm average diameter were synthesized by the standard Turkevich method, which is also known as the citrate reduction method.^38^ First, all glass materials and magnetic fish were washed with aqua regia solution (HNO_3_/3HCl) and dried under vacuum. 80 mg of gold(III) chloride trihydrate (HAuCl_4_·3H_2_O) in 200 mL deionized water (dH_2_O) was boiled with continuous stirring at 1000 rpm. Upon rapid addition of 228.22 mg of sodium citrate in 20 mL of dH_2_O to the boiling HAuCl_4_ solution, the solution turned into a reddish color. The resulting solution was shaken for another 15 min at the same speed. After cooling down at room temperature, the colloidal suspension was characterized.

The concentration of synthesized AuNPs suspension was determined by Beer–Lambert’s law.^39^ For this, the AuNPs suspensions diluted in dH_2_O (1:2, 1:4, 1:8, and 1:16 v/v) were scanned from 200 to 800 nm by UV/vis spectrometer. Their surface plasmon resonance (SPR) absorptions were recorded, and the concentration of the colloidal solution was calculated by use of these recorded values. Additionally, the number of AuNPs in 1 mL of suspension was determined via a formula proposed by Haiss et al., which was dependent on absorbance values of AuNPs at 450 nm.

### 2.2 Surface Modification of AuNPs with Carbohydrates

#### 2.2.1 Thiolation of Carbohydrates

In order to conjugate carbohydrates to AuNPs, the carbohydrates needed to be functionalized with thiol groups to enable the Au–S bond. Therefore, the carbohydrates were thiolated with sufficient yields by Lawesson reagent, which only thiolates C1 of carbohydrates.^40^ A 500 mg of carbohydrate and the Lawesson reagent (1.2 mol equiv of carbohydrate) were dissolved in 30 mL of 1,4-dioxane in a three-neck round-bottom flask. The resulting mixture was stirred at 110 °C for 48 h without any intervention under argon gas (the reaction setup image is given in Figure S1). The cooled reaction mixture was filtered through filter paper using 20 mL of 1,4-dioxane. The filtered mixture was concentrated by using a rotary evaporator. The concentrated sample was dissolved in a mixture of 50 mL of dichloromethane and 80 mL water. This mixture was transferred to a separatory funnel. After the addition of a few drops of methanol, two distinct layers were visible in the funnel. While the top aqueous layer contained thiolated carbohydrates, the bottom layer included unreacted reaction residues (the purification setup image is given in Figure S2). The bottom layer was discarded. The upper aqueous phase was divided into about 10 mL fractions and frozen at −80 °C, then dried using a freeze-dryer.

#### 2.2.2 Characterization of Thiolated Carbohydrates

The thiolated carbohydrates were characterized by Fourier transform infrared (FT-IR) spectroscopy (Thermo NICOLET IS50, Massachusetts, USA) in the attenuated total reflectance (ATR) mode.

### 2.2.3 AuNPs–Carbohydrate Conjugation Process

For a direct attachment of thiolated carbohydrates on AuNPs surfaces, the modified carbohydrates were dispersed in water by adjusting the concentration to 10 mg/mL. The AuNPs suspension (10 nM, 1 mL) was mixed with 25, 50, 75, 100, 150, and 200 μL of 10 mg/mL thiolated glucose and mannose solutions and 100, 150, and 200 μL of 10 mg/mL thiolated lactose and maltose solutions in 1.5 mL Eppendorf tubes (*n* = 3). The conjugation mix was shaken overnight at room temperature on a 3D Laboratory minishaker (Biosan Multi Bio 3D, Programmable mini-shaker). In order to remove unconjugated carbohydrates and free citrate ions, the AuNPs suspensions were centrifuged at 13,000 rpm for 20 min. The AuNPs supernatants were dispersed in deionized water, and then the naked AuNPs and AuNPs–carbohydrate conjugates were characterized.

#### 2.2.4 Characterization of Naked AuNPs and AuNP–Carbohydrate Conjugates

The optical properties, morphologies, and surface chemistries of naked AuNPs and AuNPs–carbohydrate conjugates were characterized via the following techniques.

##### 2.2.4.1 Transmission Electron Microscopy

The size, shape, and uniformity of AuNPs in suspension were characterized by transmission electron microscopy (TEM). A few microliters of AuNPs suspension were dropped on standard carbon-coated copper grids and left to air-dry for 2 h. The TEM images were taken at different magnifications by using a JEOL JEM 100C transmission electron microscope with a 70 μm lens operating at 100 kV and with 2.0 point-to-point resolution.

##### 2.2.4.2 UV/Vis Spectrometry

To monitor optical properties, the synthesized AuNPs and AuNPs–carbohydrate conjugates (1:10 diluted in dH_2_O) were characterized by a UV/vis spectrometer (Lambda 25, PerkinElmer, Foster City, CA, USA). The characteristic SPR absorption of the colloidal suspensions was obtained over the range 200 to 800 nm using a 1 cm path length quartz cuvette.

##### 2.2.4.3 Dynamic Light Scattering

The hydrodynamic sizes and zeta potentials of naked AuNPs and AuNP–carbohydrate conjugates were determined by dynamic light scattering (DLS) (ZetaSizer Nano ZS, Malvern Instruments, Malvern, UK). The 1:10 diluted AuNPs suspensions were measured in polystyrene cuvettes and in disposable capillary cuvettes. The measurements were applied with a 173° scattering angle using a 4 mW He–Ne laser at room temperature.

##### 2.2.4.4 Agarose Gel Electrophoresis

The density of carbohydrates on AuNPs in suspension was monitored by 1% agarose gel electrophoresis. In order to get condensed samples, the naked AuNPs and AuNP–carbohydrate conjugate suspensions were centrifuged at 13,000 rpm for 20 min. The red pellets were washed with dH_2_O at the same speed for the same time two more time. In the last washing step, 0.98 mL supernatants were discarded, and the pellets were suspended in the remaining 20 supernatants by soft vortexing. The condensed colloidal samples were loaded into the wells of a 1% agarose gel, which was prepared by melting 80 mg of agarose powder in 80 mL of 1× TAE buffer solution (40 mM Tris-acetate and 1 mM EDTA) by microwaving and letting it to solidify. Since the AuNPs suspension has a reddish color, no ethidium bromide (EtBr) was added to the gel. The colloidal samples loaded on the gel were run at 100 V for 1.5 h. The white image of agarose gel was taken.

##### 2.2.4.5 FT-IR Spectroscopy

In order to identify the chemical composition of AuNPs surfaces, the naked AuNPs and AuNPs–carbohydrate conjugate suspensions were characterized via FT-IR spectroscopy (Thermo NICOLET IS50, Massachusetts, USA) in ATR mode. The naked AuNPs and AuNP–carbohydrate conjugate suspensions were centrifuged at 13,000 rpm for 20 min to remove any unbound carbohydrates. The supernatants were removed, and the pellets were suspended in 1 mL of deionized water. This washing process was repeated twice. After the last washing, the pellets were resuspended in 300 μL of deionized water. The samples were frozen at −80 °C and then dried by using a freeze-dryer (Laboratory Freeze-Dryer, C-Gen Biotech, Maharashtra). The dried samples were scanned in the transmission mode from 400 to 4000 cm^–1^ by FTIR in order to achieve a good signal-to-noise ratio.

##### 2.2.4.6 Surface-Enhanced Raman Scattering

Thanks to SPR properties of AuNPs, both naked AuNPs and AuNP–carbohydrate conjugate suspensions were characterized by surface-enhanced Raman scattering (SERS) (Renishaw inVia Reflex Raman spectrometer equipped with a high-speed encoded Streamline stage, UK), which could obtain the information about carbohydrates chemically bound on AuNPs surfaces. Both the naked AuNPs and AuNPs–carbohydrate conjugate suspensions were centrifuged at 13,000 rpm for 20 min. The red pellets were dissolved in 1 mL of deionized water, and this washing process was repeated one more time. The acquired pellets were dissolved in 100 μL of double-distilled water, then 2 μL of the suspended samples were dropped on calcium fluoride (CaF_2_) slides, and then let to dry. For each sample, at least ten areas of 10 × 10 μm^2^ with a laser spot size of 1.5 μm were mapped under a 50× objective lens microscope (Leica DM2500 upright microscope) with an 830 nm photodiode laser source with 1200 lines/mm grating by applying 4 s laser exposure and 150 mV laser power. The mapped areas were preprocessed as the subtract baseline, cosmic ray removal, and smoothing. Then, the spectra obtained from the mapped area were averaged and normalized. These averaged and normalized spectra of each colloidal sample were averaged to obtain a representative spectrum. Lastly, the representative SERS spectra of naked AuNPs and AuNPs–carbohydrate conjugates were compared, and the peak alterations on the SERS spectra were investigated.

#### 2.2.5 Cell Culture

The A549 cell line (adenocarcinoma human alveolar basal epithelial cells) was cultured in DMEM/F-12 (with 4500 mg/L) supplemented with 10% FBS, 100 units/mL penicillin, 100 μg/mL streptomycin, and 2 mM l-glutamine; the BEAS-2b cell line (human bronchial epithelial cell line) was cultured in DMEM (with 4500 mg/L) supplemented with 5% FBS, 100 units/mL penicillin, 100 μg/mL streptomycin, and 2 mM l-glutamine; and the MDA-MB-231 cell line (breast cancer cell line) was cultured in DMEM (with 4500 mg/L) supplemented with 10% FBS, 100 units/mL penicillin, 100 μg/mL streptomycin, and 2 mM l-glutamine in an incubator at 37 °C with a 5% CO_2_ humidified atmosphere. When A549 and MDA-MB-231 cells reached 90–95% confluency and BEAS-2b cells reached 75–80% confluency, they were passaged.

##### 2.2.5.1 Nanoparticle Exposure to Cells

The cells were treated with either naked AuNPs or AuNPs–carbohydrate conjugate suspensions with increasing concentrations as 0.1, 0.5, 1.0, and 2.5 nM for 24 h. Before treatment, AuNPs conjugate suspensions were washed with deionized water once by centrifuging at 13,000 rpm for 20 min. Then, 970 μL of supernatant was discarded and 950 μL of deionized water was added, so all NPs were suspended in 1 mL suspension in total. Additionally, 1 mL of 13 nm AuNPs (10 nM) and AuNPs–carbohydrate conjugate suspensions included approximately 5.37 × 10^12^ particles. The cells were incubated with 1 mL of 5.37 × 10^12^ NPs for the apoptosis necrosis assay and cell cycle determination and NP uptake studies and with 2 mL of 10.74 × 10^12^ NPs for the clonogenic assay.

##### 2.2.5.2 Cellular Uptake

Cellular uptake of the naked AuNPs or AuNPs–carbohydrate conjugates was examined using flow cytometry. A549 cells (50,000), BEAS-2b cells (42,000), and MDA-MB-231 cells (50,000) were seeded in each well of 24-well plates (*n* = 3) and incubated at 37 °C in a humidified atmosphere under 5% CO_2_ for 24 h. The PBS washed cells were exposed to 0.1, 0.5, 1.0, and 2.5 nM medium-dispersed NPs and incubated for 24 h. After incubation, the cell media on the wells and the cells detached by trypsin–EDTA were collected in the same tube and then centrifuged at 2500 rpm for 5 min. The cell pellets were suspended in 200 μL of 1× PBS and immediately analyzed using a Guava easyCyte 5 (Merck Millipore) benchtop flow cytometer. In order to demonstrate the cellular uptake of naked AuNPs and AuNPs–carbohydrates, the quadrant gate on the side scatter (SSC) vs forward scatter (FSC) plot was drawn, and the percentage of each quadrant was calculated via software. As a result, the quadrant percentages of the samples were figured out as a clustered column graph analyzed with two-paired Student’s *t*-tests statistically in comparison to negative control cells. The samples with *p* ≤ 0.05 were marked with a one-star sign (*), with *p* ≤ 0.01 were marked with two-star signs (**), and with *p* ≤ 0.001 were marked with three-star signs (***).

##### 2.2.5.3 Apoptosis/Necrosis Assay

To determine the rate of apoptotic and necrotic cells of the cell population upon naked AuNPs and AuNPs–carbohydrate exposure, the Annexin V-FITC apoptosis and necrosis detection kit from Calbiochem (Merck Millipore) was utilized according to the manufacturer’s instructions. A549 cells (50,000), BEAS-2b cells (42,000), and MDA-MB-231 cells (50,000) were seeded into 24-well cell culture plates (*n* = 3) and incubated for 24 h in the incubator at 37 °C with a 5% humidified atmosphere. After 24 h, the PBS washed cells were treated with either 10% DMSO as positive control or 0.1, 0.5, 1.0, and 2.5 nM medium-dispersed NPs, and incubated for 24 h. Upon NP exposure, the cell culture media and the cells detached by trypsin–EDTA were collected into 1.5 mL Eppendorf tubes and were agitated at 2500 rpm for 5 min at 4 °C. The cell pellets were dispersed in 1× PBS. Based on manufacturer’s instructions, the dye mix was prepared as 0.5 μL of Annexin V-FITC reagent and 1 μL of PI reagent per sample was added into 200 μL of 1× binding buffer. After washing the cells with 1× PBS, one negative control was not stained with any dye to analyze unstained cells, one negative control was stained with only Annexin V-FITC in order to set up green detector voltage, one negative control was stained with only PI to adjust the red detector voltage, and finally the other negative control samples, positive control samples, and NP-treated samples were stained with both dyes in 200 μL of 1× binding mix for the purpose of apoptosis and necrosis detection. All cells were incubated with dyes in the dark for 15 min. The samples were kept at 4 °C until analysis. For each sample, 20,000 cells were counted and analyzed by using a Guava easyCyte 5 (Merck Millipore) benchtop flow cytometer. According to the calculation of the quadrant ratio of cell population by software, the results were drawn as a 2-D stacked column graph.

##### 2.2.5.4 Clonogenic Assay

To observe the survival ability of cells after NP exposure, their colony formation ability was visualized by the clonogenic assay. For this assay, 100 cells for A549, BEAS-2b, and MDA-MB-231 cell lines were seeded into each well of 6-well plates (*n* = 3) and waited for single cell’s attachment for 24 h at 37 °C in a 5% CO_2_ humidified incubator. After 24 h, the cells were treated with 2 mL of either 10% DMSO as positive control or 0.1, 0.5, 1.0, and 2.5 nM medium-dispersed naked AuNPs and AuNPs–carbohydrate conjugates. A549 and MDA-MB-231 cells were incubated for 7 days, whereas BEAS-2b cells were incubated for 10 days in a humidified incubator until each seeded single cell formed colonies including at least 50 cells. During 7–10 days of incubation in the incubator, the media in wells was not changed, and the plates were not moved anywhere to create a steady condition. In the final days of incubation, the colonies of negative controls were observed under a microscope, and the cells in each colony were counted. The incubation was stopped when the number of cells in colonies was at least 50. The media in the wells was removed, and the colonies were stained with crystal violet by incubating with the dye for 15 min. Then, the dye was taken away, and the plates were washed with water until all dye was removed from the plate. The washed plates were left to dry. The colonies were counted. The results were drawn as a clustered column graph and statistically analyzed with two-paired Student’s *t*-test to investigate the survival ability of cells exposed to increasing concentrations of NPs in comparison to negative control cells by forming colonies. The samples with *p* ≤ 0.05 were marked with one star sign (*), with *p* ≤ 0.01 marked with two-star signs (**), and with *p* ≤ 0.001 marked with three-star signs (***).

##### 2.2.5.5 Cell Cycle Evaluation

To analyze the cell cycle progression of A549, BEAS-2b, and MDA-MB-231 cells upon exposure to naked AuNPs and AuNPs–carbohydrate conjugates by flow cytometry, A549 cells (50,000), BEAS-2b cells (42,000), and MDA-MB-231 cells (50,000) were seeded into each well of 24-well plates (*n* = 3) and incubated for 24 h in an incubator at 37 °C with a 5% CO_2_ humidified atmosphere. After cell attachment, the cells were treated with 0.1 μM colchicine as a positive control and 0.1, 0.5, 1.0, and 2.5 nM of medium-dispersed NPs for 24 h. At the end of 24 h of incubation, the cell culture media in the wells and the cells detached by trypsin–EDTA were collected into the same 1.5 mL Eppendorf tubes and centrifuged at 2500 rpm for 5 min at 4 °C. The cell pellet was suspended in 1× PBS and centrifuged one more time at the same speed and time. The cell pellets were fixed with 500 μL of 70% ice-cold ethanol (v/v, ethanol in water) by gently mixing, and then the fixed cells were kept at −20 °C at least overnight. After washing the fixed cells with 1× PBS, the cell pellet was dispersed in 500 μL of 0.1% ice-cold Triton X-100 (v/v, Triton X-100 in 1× PBS) and incubated for 20 min at room temperature. After permeabilization with Triton X-100, the cells were agitated, and then the cell pellet was suspended in 200 μL of 100 μg/mL of RNase solution (v/v, RNase solution in 1× PBS) and incubated for 30 min at 37 °C to prevent the attachment of propidium iodide (PI) to RNAs, which gave positive wrong results. Lastly, PI staining was carried out as the cells were stained with 1 μg/mL for 15 min in the dark, except for one negative control, which was called unstained. Then, their cell cycle progression was analyzed by the red width vs red area plot using the flow cytometry software. The cell-cycle phases, G0/G1, S, and G2/M, were adjusted by considering those of negative and positive controls. According to the calculation of cell-cycle phase percentages by software, the results were represented in a 2-D stacked column graph.

## 3 Results

### 3.1 AuNPs Synthesis and Characterization

**Figure 1:**
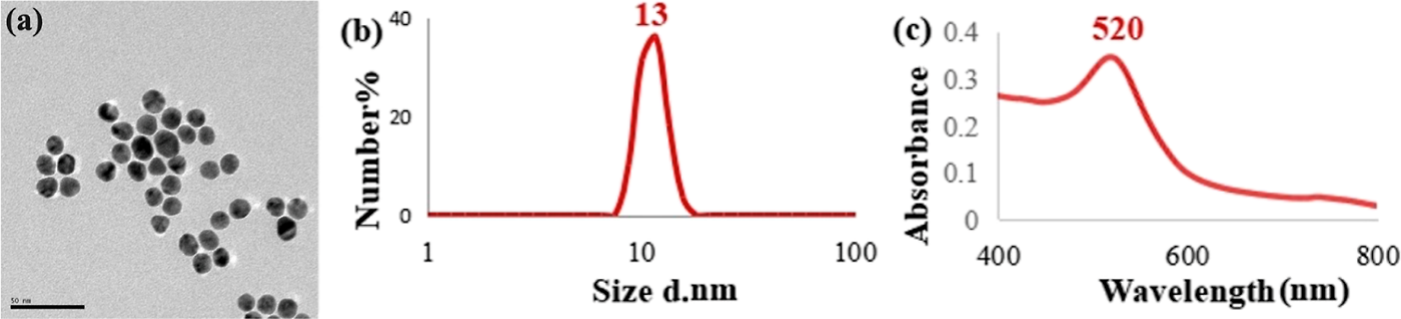
Characterization of a colloidal suspension containing 13 nm AuNPs. (a) TEM image, (b) UV/vis spectrum, and (c) DLS spectrum.

The synthesized AuNPs were characterized by TEM, UV/vis spectroscopy, and dynamic light scattering (DLS) and are shown in Figure 1. The TEM images showed that the AuNPs were of 13 nm average diameter. Additionally, UV/vis and DLS spectra were characteristic of the AuNP colloidal suspension synthesized with the method.

The concentration of the naked AuNPs suspension was determined by Beer–Lambert’s law, and the total number of AuNPs in 1 mL suspension was calculated by using a formula suggested by Haiss et al.^39^ Based on the calculations given in the Supporting Information, the concentration was found as 10 nM, and the total number of AuNPs in 1 mL of suspension was determined as 5.37 × 10^12^. In addition, since the color of AuNPs–carbohydrate suspensions was darker than the naked AuNP suspension (shown in Figures S3–S6) and the determination of AuNPs concentration by UV/vis spectroscopy depends on the color of the suspension, the concentration of AuNPs–carbohydrate suspensions was assumed as much as that of the naked AuNPs suspension.

### 3.2 Surface Modification of AuNPs with Carbohydrates

**Figure 2:**
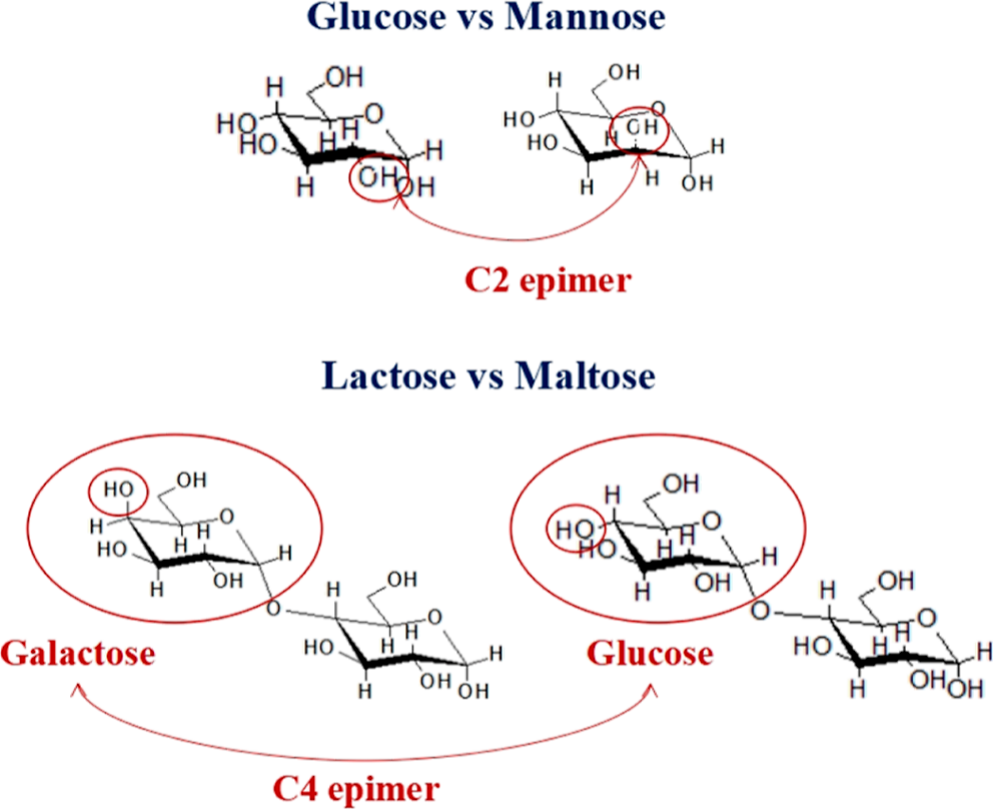
Scheme of hydroxyl group orientation differences between glucose and mannose and free monosaccharide differences between lactose and maltose after conjugation.

As mentioned above, carbohydrates have been used for the surface modification of AuNPs since they are major targeting molecules due to their unique molecular characteristics and actions in living systems.^41^ Many types of carbohydrates have been conjugated with AuNPs to study carbohydrate–carbohydrate and carbohydrate–protein interactions ex vivo and in vivo or in vitro.^24,42^ Carbohydrates cannot link AuNPs surfaces directly, thus the linkage between carbohydrates and AuNPs can be established through a thiol group –SH.^43^ In this study, it was aimed to conjugate AuNPs with glucose, mannose, lactose, and maltose via Au–S bond formation. The reason they were chosen was that glucose and mannose were epimers at the second carbon, and the free units of lactose and maltose were galactose and glucose, which were epimers at C4, as shown in Figure 2. The mentioned differences would be helpful to investigate the cellular response of living cells to subtle surface chemistry changes on NP surfaces.

#### 3.2.1 Thiolation of Carbohydrates and Their Characterization

In order to attach carbohydrates to AuNPs, they were thiolated by Lawesson reagent to create the C–S bond at the first carbon and then the AuNPs surfaces were coated to obtain the thiol-modified carbohydrates, as shown in Figure S7.^40^ The thiolated carbohydrates were characterized by FTIR. The comparative FTIR spectra of unmodified and thiolated carbohydrates are given in Figure S8. The weak peaks around 2550–2650 and 1650 cm^–1^ in the spectra are attributed to –S–H and C–S vibrations, respectively. However, a broad peak at around 3200 cm^–1^ originating from –O–H bond vibrations is observed only on the spectra of unmodified carbohydrates.^44^ The comparison of the FTIR spectra suggests that thiol modification was successful.

#### 3.2.2 Conjugation of AuNPs with Carbohydrates and Their Characterization

**Figure 3:**
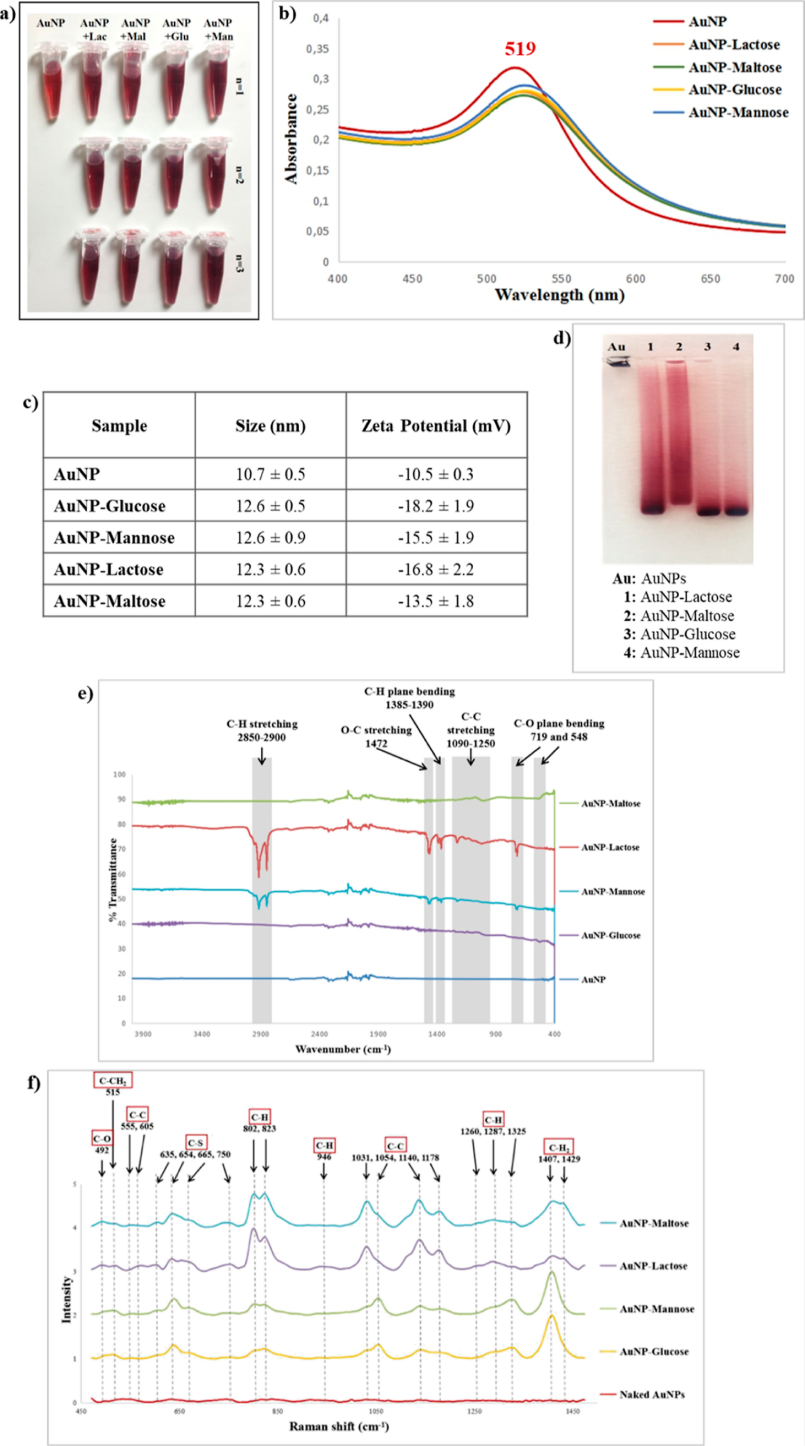
Characterization of the naked AuNPs and AuNPs–carbohydrate conjugates. (a) White light image, (b) UV/vis spectra, (c) hydrodynamic sizes and zeta potentials, (d) agarose gel, € FTIR spectra, and (f) SERS spectra.

Based on optimization studies given in Figures S9–S14, 1 mL of 10 nM 13 nm AuNPs was conjugated with 75 μL of 10 mg/mL thiolated lactose and maltose and 150 μL of 10 mg/mL thiolated glucose and mannose solutions. The naked AuNPs and AuNPs–carbohydrate conjugates were characterized by UV/vis spectroscopy, DLS, 1% agarose gel electrophoresis, FTIR spectroscopy, and SERS. As AuNPs are plasmonic materials, they are very sensitive to the changes in the dielectric layer forming on their surfaces in their suspensions, which can be monitored from their UV/vis spectra as a shift in their SPR peak and through SERS of molecular species in close vicinity to their surfaces. It is also possible to monitor the color as result of the changes in their surface properties and aggregation status due to the wavelength shifts in scattered light as a qualitative indicator. The comparative white light images, UV/vis spectra, DLS data, gel electrophoresis image, and SERS data of suspensions of the naked AuNPs and AuNPs–carbohydrate conjugates are provided in Figure 3. From the white light images of the AuNPs suspensions, it can be seen that there is no indication of aggregation/precipitation with the addition of thiol-carbohydrate conjugates into the naked AuNPs suspensions but a slightly darker color. Figure 3b shows the evolution of the SPR peaks as monosaccharide- and disaccharide-coated samples are attached. While the suspension of the naked 13 nm AuNPs is observed at 519 nm, with the attachment of monosaccharide and disaccharide, the SPR peak is shifted to 524 and 525 nm, respectively.

The hydrodynamic sizes and zeta potentials can provide more information about the changes on the surfaces of AuNPs. Figure 3c summarizes the hydrodynamic sizes and zeta potentials of the naked AuNPs and AuNPs–carbohydrate conjugates. As is seen, the hydrodynamic size of the naked AuNPs increases with the attachment of carbohydrates. Interestingly, the hydrodynamic size of glucose and mannose conjugates is higher than the maltose and lactose conjugates. This is possibly because of the formation a stronger ionic layer over the shorter monosaccharides although the zeta potential data does not correlate with this observation. A change in zeta potentials of the mono- versus disaccharide compared to naked AuNPs is observed, but there is no clear trend possibly due to the ionic strength differences from suspension to suspension. The reason behind this can be explained with the variation in the free citrate ion interaction with –OH groups of carbohydrates during the replacement between the citrate ions and carbohydrates on the AuNPs surface, as illustrated in Figure S15. The interaction between –OH groups and citrate ions are so strong that the multiple wash procedure does not remove the citrate ions completely.

In order to evaluate the carbohydrate densities on AuNPs, the naked AuNPs and AuNPs–carbohydrate conjugates were characterized by 1% agarose gel electrophoresis, as seen in Figure 3d. The naked AuNPs precipitated whenever loaded into the gel due to the high salt content of TAE buffer. However, the conjugates ran through the gel since their surfaces are conjugated on AuNPs surfaces with enough carbohydrate density and thus they are less affected by salt ions. Additionally, monosaccharide-conjugated AuNPs ran longer distances on the gel than disaccharide-decorated ones, perhaps due to the smaller size and less ionic load on their surfaces.

For chemical evidence that carbohydrates attached to the AuNPs surfaces, the naked AuNPs and AuNPs–carbohydrate conjugates were characterized by FTIR, and their comparative FTIR spectra are shown in Figure 3e. On the FTIR spectrum of naked AuNPs, almost no spectral information is observed, as expected, because they had only citrate ions on their surfaces. On the other hand, on the spectra of AuNPs conjugates, several peaks attributed to carbohydrates, such as C–H stretching at 2850–2900 cm^–1^, O–C stretching at 1472 cm^–1^, C–H plane bending at 1385−1390 cm^–1^, C–C stretching at 1090–1250 cm^–1^, and C–O plane bending at 719 and 548 cm^–1^, are observed.^44^

The naked AuNPs and AuNPs–carbohydrate conjugates were further characterized with SERS in order to evidence those carbohydrates conjugated to the AuNPs surfaces. The comparative SERS spectra of naked AuNPs and AuNPs–carbohydrate conjugates are presented in Figure 3f. As is seen, there is no noteworthy chemical information on the SERS spectra of the naked AuNPs. However, the characteristic peaks originating from carbohydrates on the AuNPs–carbohydrate conjugates, such as C–O stretching at 492 cm^–1^, C–C stretching at 555, 605, 1031, 1054, 1140, and 1178 cm^–1^, C–S stretching at 635, 654, 665, and 750 cm^–1^, C–H stretching at 82, 823, 946, 1260, 1287, and 1325 cm^–1^, C–CH_2_ stretching at 515 cm^–1^, and C–H_2_ stretching at 1407 and 1429 cm^–1^, are observed.^45^ Furthermore, similar spectral patterns and common peak intensities are observed on the spectra of AuNPs–glucose and AuNPs–mannose and on AuNPs–lactose and AuNPs–maltose. On the spectra of AuNPs conjugated with monosaccharides, C–S stretching at 635 cm^–1^, C–C stretching at 1054 cm^–1^, C–H stretching at 1325 cm^–1^, and C–H_2_ stretching at 1407 cm^–1^ are dominant peaks. Nevertheless, C–H stretching at 802 and 823 cm^–1^ and C–C stretching at 1031, 1140, and 1178 cm^–1^ are dominant on the spectra of AuNPs decorated with disaccharides. In conclusion, the SERS spectra of the conjugates support the binding of the ligand bound to the AuNPs surfaces. Considering all of the characterization data, one can conclude that the surfaces of AuNPs are successfully functionalized with the thiolated carbohydrates.

### 3.3 Cellular Response to Carbohydrate-Conjugated AuNPs

**Figure 4:**
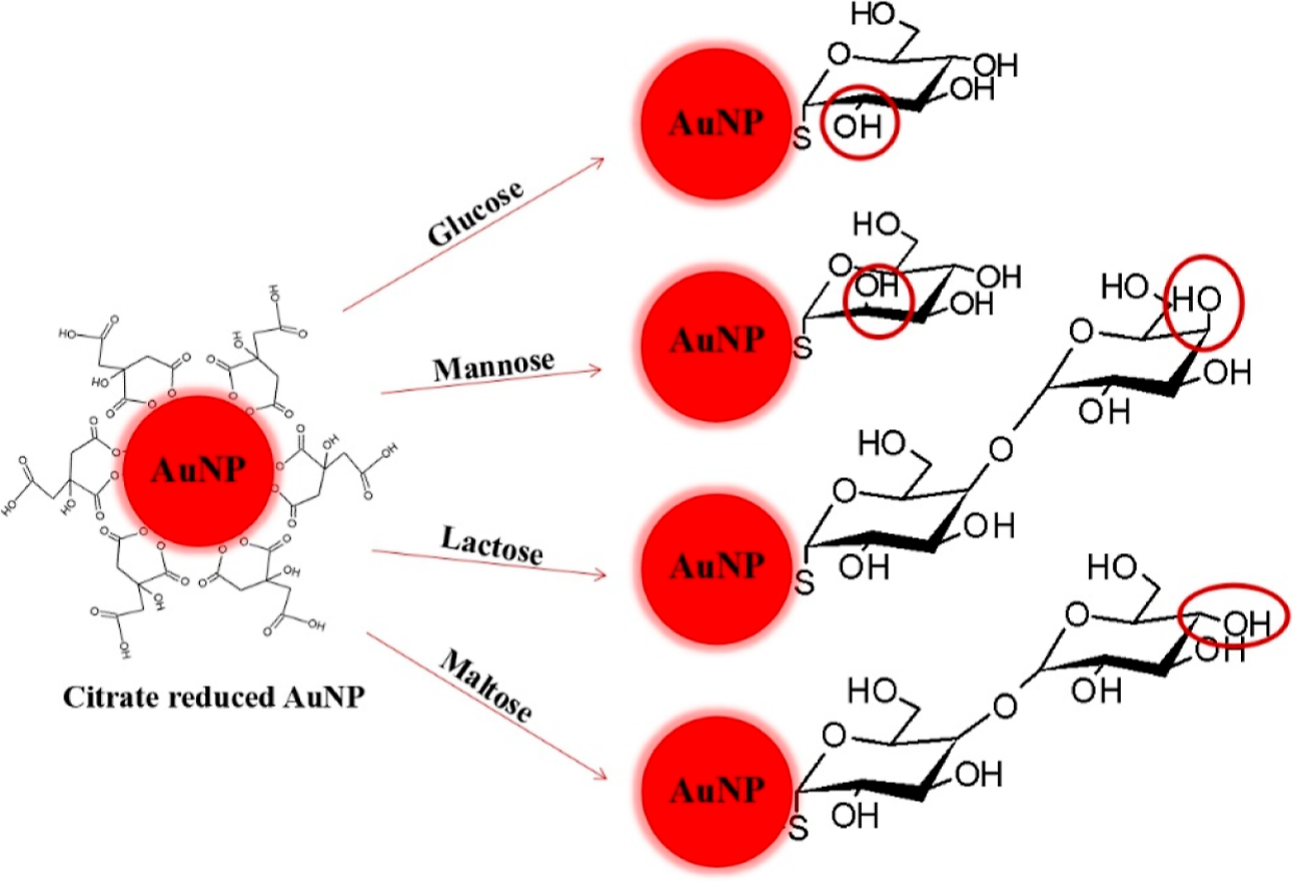
Subtle differences on AuNPs surfaces after functionalization with thiolated glucose, mannose, lactose, and maltose.

Figure 4 shows the chemistry differences aimed at generation on the AuNPs surfaces. Glucose and mannose are epimers at C2, and the free units of lactose, maltose, galactose, and glucose are epimers at C4.

#### 3.3.1 Cellular Uptake Studies

**Figure 5:**
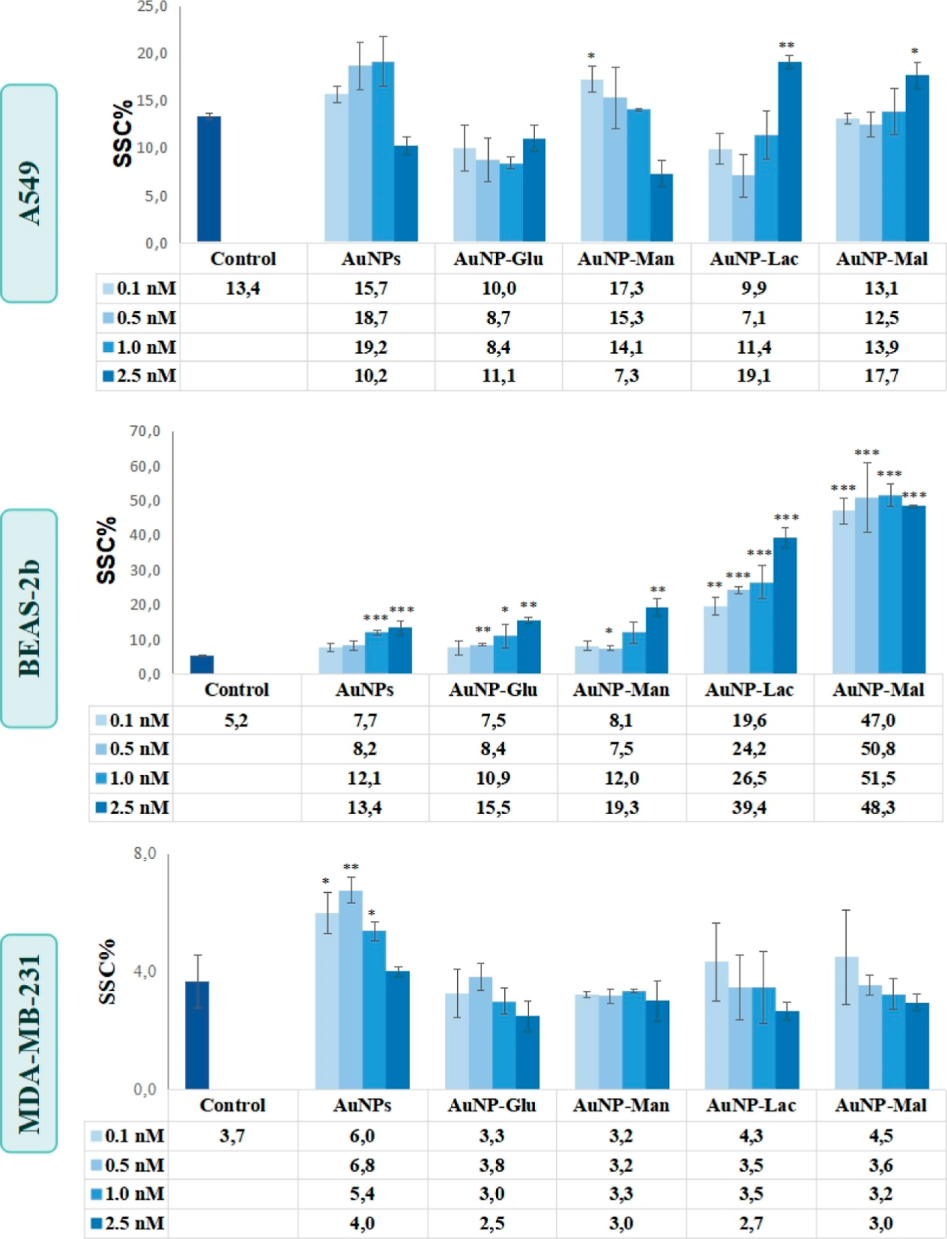
SSC graphs of A549, BEAS-2b, and MDA-MB-231 cells treated with 0.1, 0.5, 1.0, and 2.5 nM naked 13 nm AuNPs and glucose-, mannose-, lactose-, and maltose-functionalized AuNPs. Statistically significant changes compared to negative control cells were calculated by two-paired Student’s *t*-test and marked with stars, * for *p* ≤ 0.05, ** for *p* ≤ 0.01, and *** for *p* ≤ 0.001.

The cellular uptake of nanoparticles can be monitored via flow cytometry because the NPs taken up by cells or attached to their cell membrane increase the granulation of cell, which was detected by an increase on SSC light.^46^ SSC graphs of A549, BEAS-2b, and MDA-MB-231 cells treated with 0.1, 0.5, 1, and 2.5 nM naked 13 nm AuNPs, AuNPs–glucose, AuNPs–mannose, AuNPs–lactose, and AuNPs–maltose conjugates are given in Figure 5. The negative control cells of A549, BEAS-2b, and MDA-MB-231 cells showed approximately 13, 5, and 4% granulation, respectively.

A549 cells internalized disaccharide-modified AuNPs at 2.5 nM and mannose-conjugated ones at 0.1 nM. The free glucose and galactose (C4 epimer) on disaccharides and mannose (C2 epimer) on the surface allowed the internalization of their conjugates. Surprisingly, the uptake of glucose-conjugated AuNPs in A549 cells was not considerable, despite the high avidity of cancerous cells for glucose. This could be due to the presence of a protein corona layer on the surface of the modified AuNPs. As the NPs are added into cell culture media, a layer of tightly or loosely bound proteins and perhaps other ionic and molecular species are formed depending on the surface chemistry and charge. Besides, the orientation of proteins initially bound to the NPs surface influences the molecular composition of the protein corona, which might eventually affect the cellular uptake. This suggests that the role of t–e −OH orientation at C2 and C4 of saccharide can have been a factor for the uptake of the AuNPs in A549 cells. It is possible that with the difference in –OH orientation, one protrudes toward the surface, while the other towards the NP surface, altering the hydrophilicity and hydrogen bonding ability with the molecular and ionic species in the cell culture medium. Furthermore, BEAS-2b cells significantly internalized all AuNPs, especially the AuNPs–maltose conjugates. The disaccharide-modified AuNPs penetrated BEAS-2b cells more than the monosaccharide-tailored ones. The free glucose of maltose and free galactose (C4 epimers) of lactose increased the uptake of the enzyme into BEAS-2b cells. Again, t–e −OH orientation at C4 of free glucose may play an important role in the AuNPs uptake in BEAS-2b cells because all exposed concentrations of AuNPs–maltose caused the same granulation. Finally, MDA-MB-231 cells internalized only the naked AuNPs, whereas the coating of AuNPs surfaces with carbohydrates inhibited AuNPs internalization.

#### 3.3.2 Apoptosis/Necrosis Assay

**Figure 6:**
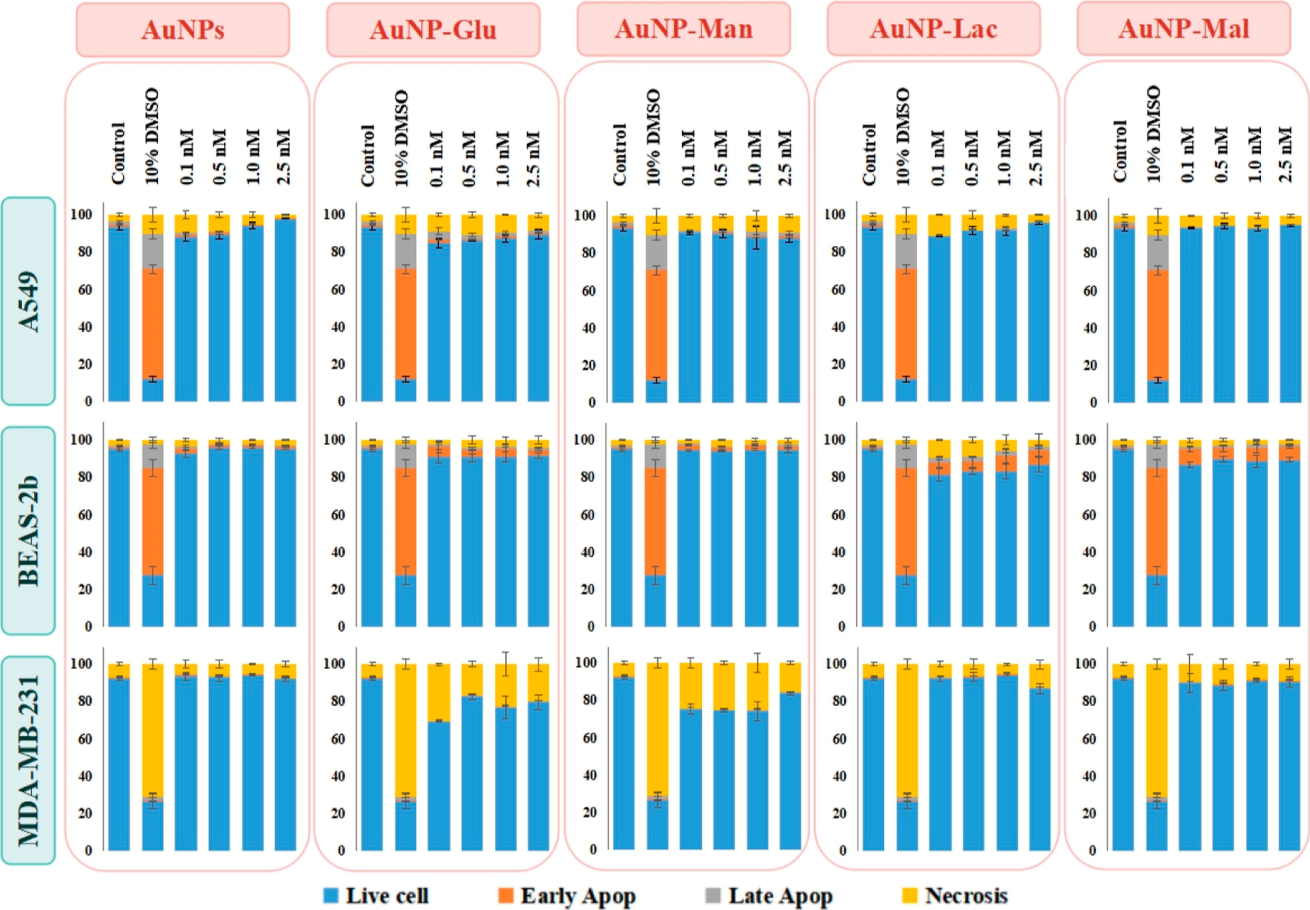
Apoptosis/necrosis assay result of A549, BEAS-2b, and MDA-MB-231 cells treated with 0.1, 0.5, 1.0, and 2.5 nM of naked 13 nm AuNPs and glucose-, mannose-, lactose-, and maltose-functionalized AuNPs. The positive control was 10% DMSO.

The apoptosis/necrosis assay result of A549, BEAS-2b, and MDA-MB-231 cells treated with 0.1, 0.5, 1.0, and 2.5 nM of naked 13 nm AuNPs and glucose-, mannose-, lactose-, and maltose-modified AuNPs is given in Figure 6. The positive control was 10% DMSO, and apoptosis was induced for A549 and BEAS-2b cells and necrosis for MDA-MB-231 cells.

A549 cells exposed to either naked AuNPs or AuNPs–carbohydrate conjugates showed different cytotoxic profiles. The naked AuNPs in the 0.1–0.5 nM dosage range induced necrosis in A549 cells. The monosaccharide-conjugated AuNPs demonstrated concentration-dependent cytotoxicity as a lower dose caused higher cytotoxicity in AuNPs–glucose-exposed cells, while higher doses of AuNPs–mannose resulted in higher toxicity in A549 cells. In other words, the orientation of −OH at C2 directly affects the toxicological behavior of the A549 cells. Additionally, it can be claimed that 0.1 nM and lower dosages of the AuNPs–glucose conjugate may induce apoptotic pathways in A549 cells. When considering the disaccharide-conjugated AuNPs exposure, AuNPs–lactose at 0.1–0.5 nM dose range induced necrosis in approximately 5% of the cells of the population, whereas no significant cytotoxicity was investigated in the cells treated with AuNPs–maltose. Again, one can conclude that the −OH orientation of free galactose (C4 epimer) on AuNPs–lactose may stimulate necrosis in A549 cells at lower concentrations.

AuNPs–glucose, AuNPs–lactose, and AuNPs–maltose conjugates resulted in apoptosis in BEAS-2b cells, whereas AuNPs–mannose showed no significant cytotoxicity. The glucose-coated AuNPs at all concentrations induced apoptosis in approximately 5% BEAS-2b cells in the population, while no remarkable toxicity was observed in the cells after mannose-conjugated AuNPs (C2 epimer). Based on the results, it can be said that the −OH orientation at C2 of glucose may have a critical role in the apoptotic pathways of BEAS-2b cells. The disaccharide-conjugated AuNPs created higher toxicity in BEAS-2b cells compared to the monosaccharide-conjugated ones. Especially, AuNPs–lactose conjugates at 0.1 nM dose reduced the live cell percentage up to 80%. It should be noted that the free galactose of lactose (C4 epimer) should play a critical role in the toxicological profile of BEAS-2b cells. Additionally, the number of free glucose molecules on AuNPs surfaces influenced the toxicity percentage as AuNPs–glucose and AuNPs–maltose induced approximately 5 and 10% apoptosis in BEAS-2b cells, respectively. With this result, it is clear that the presence of a flexible glucose unit on the AuNPs surface increases the cytotoxicity in BEAS-2b cells.

The monosaccharide-conjugated AuNPs induced remarkable necrosis in MDA-MB-231 cells compared to the disaccharide-tailored AuNPs. Approximately 30–35% cells of the population went to necrosis after exposure to the AuNPs–glucose and AuNPs–mannose with a pattern. AuNPs–glucose conjugates at a 0.1 nM dose induced the highest cytotoxicity. However, AuNPs–mannose conjugates at a 2.5 nM dose increased the cell viability. Indeed, the −OH orientation at C2 of monosaccharides and the applied dose disparately affected the cell viability of the MDA-MB-231 cells. AuNPs–lactose at 0.1–2.5 nM dose and AuNPs–maltose at 2.5 nM dose elicited low cytotoxicity, and necrosis was induced in approximately 1–2% more MDA-MB-231 cells. Based on this result, it can be concluded that the –OH orientation at C4 of disaccharides had a smaller role in necrotic pathways of MDA-MB-231 cells, while the orientation at C2 of monosaccharides had a critical role in these pathways.

#### 3.3.3 Clonogenic Assay

**Figure 7:**
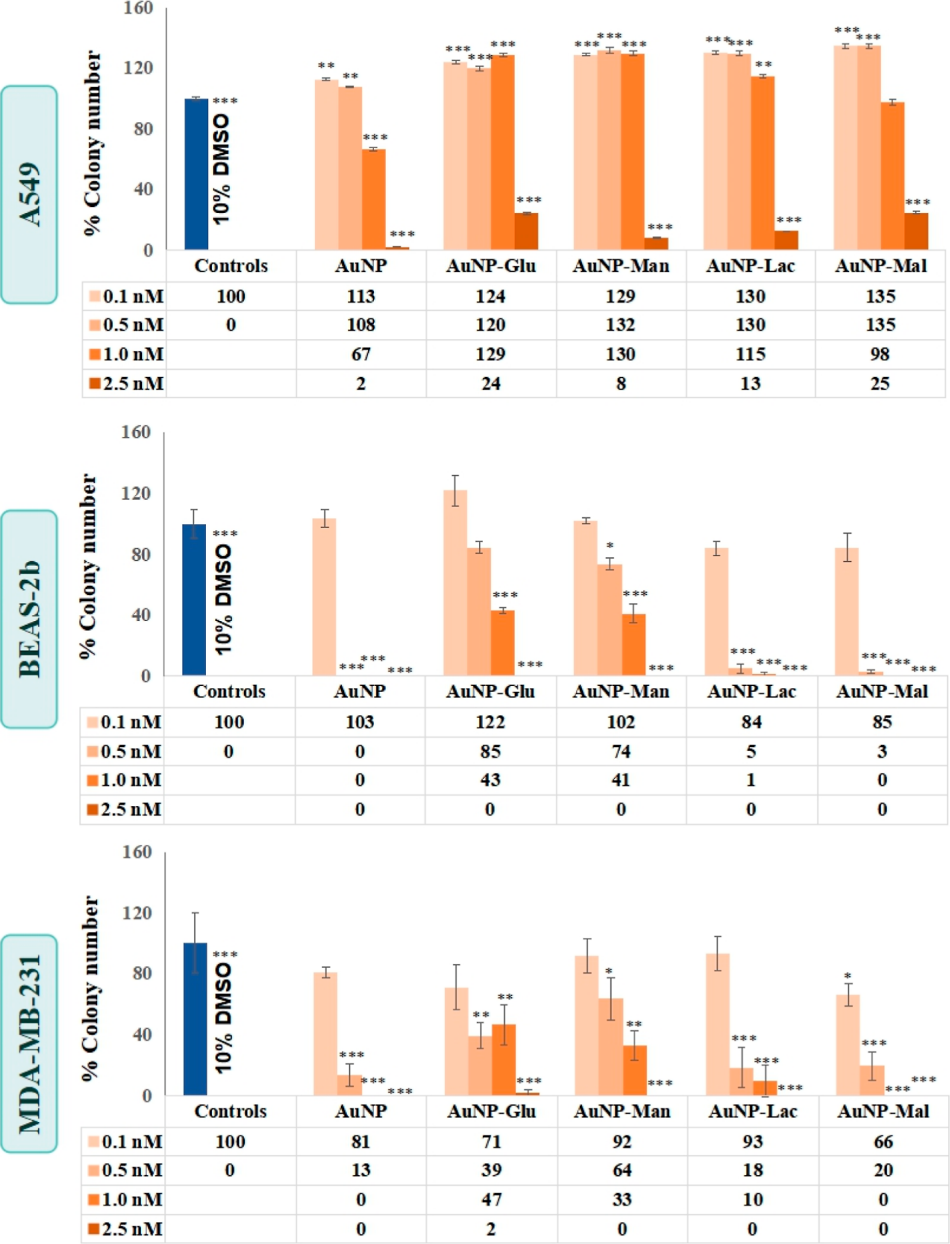
Clonogenic assay result of A549, BEAS-2b, and MDA-MB-231 cells treated with 0.1, 0.5, 1.0, and 2.5 nM of naked 13 nm AuNPs and glucose-, mannose-, lactose-, and maltose-functionalized AuNPs. The positive control was 10% DMSO. Statistically significant changes compared to negative control cells were calculated by two-paired Student’s *t*-test and marked with stars, * for *p* ≤ 0.05, ** for *p* ≤ 0.01, and *** for *p* ≤ 0.001.

As a last toxicity assay, the clonogenic cell survival assay, which is a sensitive technique, was performed to examine the colony formation ability of a single cell under the treatment conditions. To investigate cell survival upon NPs exposure is an essential phenotypic measurement to get information whether exposed NPs induced or prevented toxicity.^47^ It was expected that the colony formation ability or formed cell colonies should decrease in parallel to increasing concentration of NPs. Thanks to the clonogenic cell survival assay, long-term cytotoxicity of NPs can be evaluated. Therefore, the clonogenic cell survival assay for A549, BEAS-2b, and MDA-MB-231 cells treated with increasing concentrations of either naked 13 nm AuNPs or glucose-, mannose-, lactose-, and maltose-conjugated AuNPs were performed, and the results are given in Figure 7. 10% DMSO was used as a positive control, and no colony formation was seen in all cell types in its presence.

A549 cells exposed to the naked AuNPs and carbohydrate-conjugated AuNPs can be referred to as clonogenic since the seeded single A549 cells proliferated and produced a colony including a large number of cells to enable their integrity. Based on the results, A549 cells had the ability to survive under the conditions of 0.1–1.0 nM of either naked AuNPs or carbohydrate-conjugated AuNPs, and only 2.5 nM doses blocked the colony formation of the single A549 cells when compared to control untreated cells. These results were consistent with the cytotoxic results given in Figure 6. As a conclusion, –OH orientation difference at C2 and C4 of mono- and disaccharides on AuNPs surfaces did not have a significant effect on the colony formation of A549 cells.

BEAS-2b cells treated with just 0.1 nM of all AuNPs–carbohydrate conjugates were called clonogenic as their colony formation ability was similar to negative control cells. Moreover, the BEAS-2b cells exposed to the higher concentrations of AuNPs–lactose and AuNPs–maltose were visualized as unable to divide or go through one or two mitoses. On the other hand, BEAS-2b cells treated with 0.5–1.0 nM doses of AuNPs–glucose and AuNPs–mannose could survive by forming colonies. These colony formation results were also consistent with the cytotoxic results given in Figure 6. It can be concluded that the –OH orientation at C2 of monosaccharides enabled them to survive and reproduce single BEAS-2b cells, whereas the −OH orientation at C4 of disaccharides may block the colony formation of BEAS-2b cells.

MDA-MB-231 cells as a breast carcinoma cell line showed different colony formation behavior compared to A549 cells, the lung cancer line. MDA-MB-231 cells exposed to 0.1 nM of AuNPs–glucose, AuNPs–mannose, and AuNPs–lactose were designated as clonogenic since the single MDA-MB-231 cells at that treatment reproduced and created a colony as much as the untreated control. On the other hand, single MDA-MB-231 cells could not proliferate enough to form a colony, and the total colony number under AuNPs–maltose treatment was significantly low in comparison to the untreated control. Considering this result, it can be said that the free glucose on maltose-conjugated AuNPs at 0.1–2.5 nM concentration may block the survival pattern in MDA-MB-231 cells, but its C4 epimer galactose on AuNPs–lactose at 0.1 nM dose could induce proliferation. Furthermore, cells treated with monosaccharide-conjugated AuNPs had the ability to survive more than those exposed to disaccharide-conjugated AuNPs. In this case, it was seen that the colony formation ability of single MDA-MB-231 cells under AuNPs–carbohydrate conjugate exposure was more influenced by the number of saccharides on AuNPs surfaces than C2 epimers. These results were also consistent with cytotoxicity results given in Figure 6.

#### 3.3.4 Cell Cycle Evaluation

**Figure 8:**
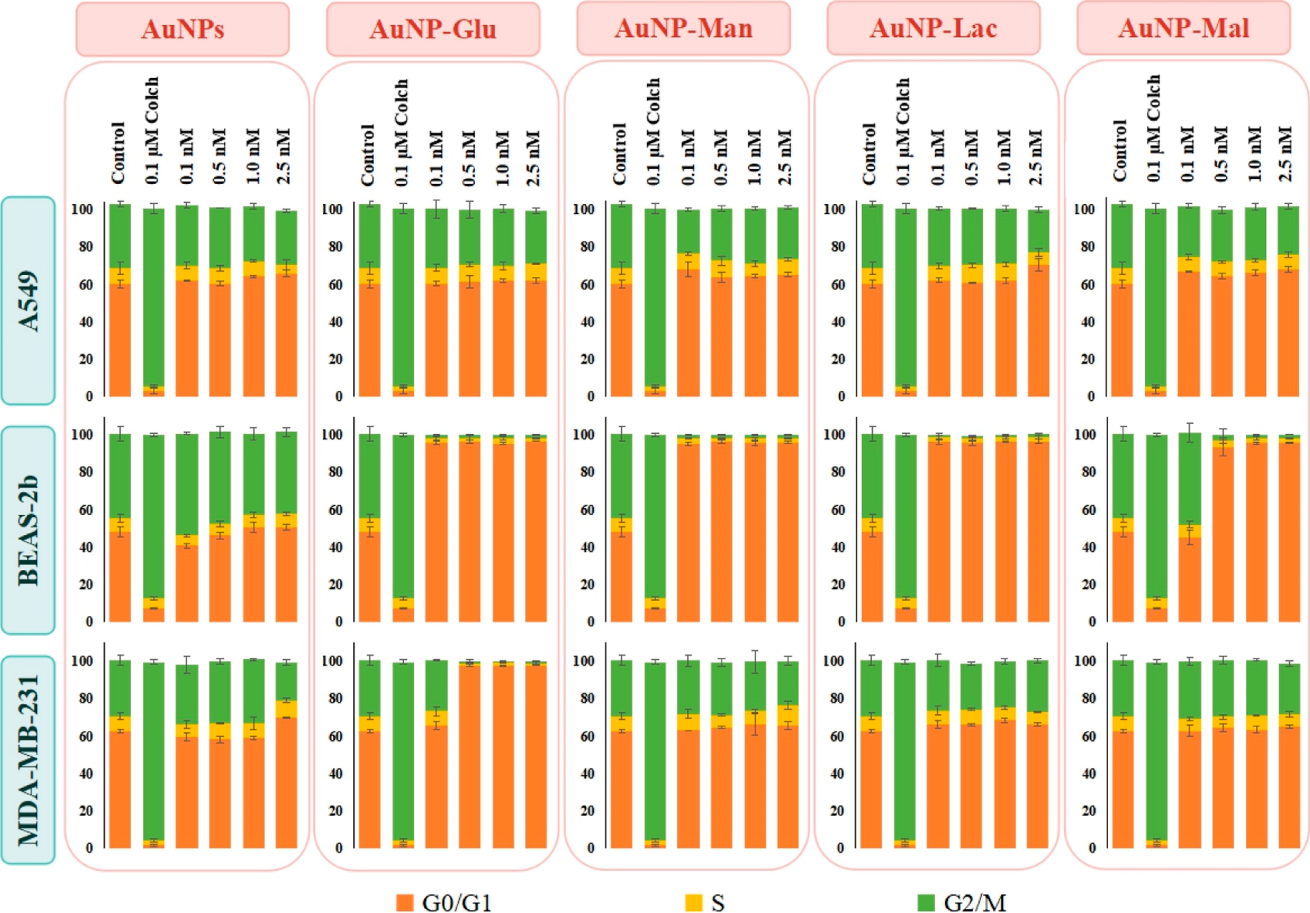
Cell cycle progression of A549, BEAS-2b, and MDA-MB-231 cells treated with 0.1, 0.5, 1.0, and 2.5 nM of naked 13 nm AuNPs and glucose-, mannose-, lactose-, and maltose-functionalized AuNPs. The positive control was 0.1 μM colchicine.

Only cytotoxicity evaluation is not sufficient to understand the cellular response to the NPs surface chemistry. The concentration of NPs applied could not result in cytotoxicity but can affect cell cycle progression and related severe pathways. The cell cycle progression of A549, BEAS-2b, and MDA-MB-231 cells treated with increasing concentrations of either naked 13 nm AuNPs or glucose-, mannose-, lactose-, and maltose-functionalized AuNPs is seen in Figure 8. 0.1 μM colchicine was used as a positive control because it blocks cells at the G2/M phase, and it arrested 85–90% of all cell types at the G2/M phase.

The cell cycle of A549 cells was blocked at the G0/G1 phase after incubation with 0.1 nM AuNPs–mannose and 2.5 nM AuNPs–lactose, whereas no significant change in cell cycle progression was observed after treatment with any dose of naked AuNPs, AuNPs–glucose, and AuNPs–maltose. These alterations in the cell cycle of A549 cells were uptake related. In other words, the internalized AuNPs conjugates stimulated G0 and G1 phase blockage in A549 cells. Additionally, –OH orientations at C2 and C4 of glucose did not affect the cell cycle of A549 cells; however, those of mannose (C2 epimer) and free galactose on lactose (C4 epimer) resulted in cell cycle arrest in A549 cells.

Gold glyconanoparticles designed in this study dramatically affected BEAS-2b cell cycle progression. Approximately 90% of BEAS-2b cells were arrested at the G0/G1 phase, except for the cells treated with 0.1 nM of AuNPs–maltose. Based on the result, it was deduced that the free glucose on AuNPs–maltose at only 0.1 nM dosage did not cause any significant effect on the cell cycle progression of BEAS-2b cells.

The cell cycle of MDA-MB-231 cells was arrested by only monosaccharide-conjugated AuNPs. The AuNPs–glucose at 0.5–2.5 nM and AuNPs–mannose at 2.5 nM caused G0/G1 phase arrest in MDA-MB-231 cells. In addition, the arrest caused by AuNPs–glucose was very dramatic since approximately 95% of MDA-MB-231 cells in the population were blocked at the G0/G1 phase. Although AuNPs–glucose and AuNPs–mannose conjugates induced necrosis in MDA-MB-231 cells, only AuNPs–glucose resulted in a dramatic cell cycle arrest in the cells. This indicated that −OH orientation at C2 of monosaccharides can interact with cyclin-dependent kinase (CDK) pathways and also that the concentration of the AuNPs conjugates specifically managed the cell cycle arrest.

## 4 Discussion

In this study, we systematically investigated the cellular response to –OH group orientation differences on carbohydrate-coated nanoparticle surfaces. Four custom chosen carbohydrates, glucose, mannose, lactose, and maltose, were thiolated with Lawesson reagent to thiolate only the first carbon in order to investigate cellular responses to −OH group orientation at the second carbon of monosaccharides and the fourth carbon of disaccharides, and they were then conjugated with the spherical AuNPs with 13 nm diameter to obtain a monolayer carbohydrate surface. The therapeutic effects of C2 epimerism of monosaccharides and C4 epimerism of disaccharides on AuNPs were screened on A549, BEAS-2b, and MDA-MB-231 cells. This study demonstrated subtle differences on carbohydrates as the −OH orientation result in NP concentration-, surface chemistry-, and cell line-dependent cellular responses. Additionally, the number of saccharides on AuNPs surfaces also dramatically influenced the cellular uptake of gold glycol nanoparticles and cytotoxicity and cell cycle progression of cell lines.

The cellular response of A549 cells exposed to gold glycol nanoparticles with subtle −OH orientation differences was significantly affected by C2 and C4 epimerism. The 0.1 nM mannose (C2 epimer)- and lactose (free unit galactose, C4 epimer)-conjugated AuNPs reduced the cell viability of A549 cells by triggering different death mechanisms. That is, C2 epimerism induced apoptosis, while C4 epimerism stimulated necrosis. Moreover, C2 epimerism not only caused toxicity in A549 cells but also resulted in cell cycle arrest. Approximately 5–10% of A549 cells exposed to 0.1 nM AuNPs–mannose and 2.5 nM AuNPs–lactose were arrested at the G0/G1 cell cycle. It can be concluded that since AuNPs–mannose at a very low dose of 0.1 nM induced apoptosis and showed a significant antiangiogenic effect in A549 cells, and mannose can be preferred instead of glucose in the design of gold glyconanoparticles.

The cellular response of BEAS-2b cells, a healthy bronchial cell line, to −OH orientation differences on gold glyconanoparticles was associated with not only the −OH orientation at C2 or C4 but also the number of saccharides on gold glyconanoparticles designed in this study. All gold glyconanoparticles were significantly internalized by BEAS-2b cells and increased the cellular granulation, especially disaccharide-modified ones. As the same amount of granulation was monitored in the BEAS-2b cells treated with AuNPs–maltose (glucose saccharide unit), it can be said that the −OH orientation of free glucose on AuNPs–maltose can be correlated to the NPs uptake mechanism of BEAS-2b cells. Additionally, the disaccharide-conjugated AuNPs induced higher cytotoxicity in comparison to the monosaccharide-tailored ones. It can be interpreted that the −OH orientation difference at C2 and C4 can play a critical role in the toxicological behavior of BEAS-2b cells since free galactose of AuNPs–lactose and free glucose of AuNPs–maltose stimulate necrosis and apoptosis in BEAS-2b cells, respectively. Moreover, the number of glucose units on gold glyconanoparticles influenced the cytotoxicity as the higher glucose unit stimulated higher apoptosis in BEAS-2b cells. On the other hand, no remarkable cytotoxicity was observed in BEAS-2b cells exposed to AuNPs–mannose. This toxicological profile was also complementary to the colony formation ability of BEAS-2b cells, which is related to the long-term toxicity, as the monosaccharide-conjugated AuNPs showed higher survival ability by forming colonies in comparison to the disaccharide ones. In other words, the high uptake of disaccharide-conjugated AuNPs may cause low survival ability by forming colonies from single BEAS-2b cells. When considering the cell cycle progression of BEAS-2b cells after exposure to gold glyconanoparticles, only AuNPs–maltose at 0.1 nM concentration did not induce cell cycle arrest in BEAS-2b cells. Based on all these results, it was clearly seen that not only −OH orientation at C2 and C4 of gold glyconanoparticles but also the number of free glucoses on NPs surfaces influence their cellular uptake and toxicological behavior in BEAS-2b cells, and AuNPs–mannose (C2 epimer) can be highlighted as a new therapeutic agent for lung cancer by considering complementary consequences of A549 cells.

MDA-MB-231 cells as a carcinoma cell line demonstrated significant response to −OH orientation differences on gold glyconanoparticles surfaces. The monosaccharide-modified AuNPs caused severe necrosis in MDA-MB-231 cells, while the disaccharide-conjugated ones showed no remarkable cytotoxicity. With this result, it can be speculated that the –OH orientation at C4 of disaccharides on AuNPs had a fewer role in necrotic pathways of MDA-MB-231 cells, while the orientation at C2 of monosaccharides on AuNPs had a critical role in these pathways. When considering the long-term toxicity, MDA-MB-231 cells exposed to AuNPs–maltose were affected dramatically. The free glucose on AuNPs surfaces at 0.1 nM concentration blocked the colony formation ability of single MDA-MB-231 cells, and colonies were monitored at the treatment with its C4 epimer at that dose. Moreover, a dramatic cell cycle arrest at the G0/G1 phase was investigated in MDA-MB-231 cells exposed to AuNPs–glucose. It can be speculated that C2 epimerism on AuNPs–monosaccharide surfaces could play a critical role in CDK pathways. Based on all results, the response of MDA-MB-231 cells treated with four custom-designed four gold glyconanoparticles was associated with the saccharide number on NPs as monosaccharide-conjugated AuNPs demonstrated more angiogenic effect in the cells, and AuNPs–glucose at 0.5 nM concentration and more can be used as either a novel therapeutic agent or a drug delivery system for breast cancer.

## 5 Conclusions

As a conclusion, this study showed that the −OH orientation on gold glyconanoparticles surfaces is critical in cellular response and must be taken as one of the major parameters during designing of novel therapies. Also, this is the first systematic study to investigate the cellular response to subtle −OH orientation at gold glyconanoparticles, demonstrating that the surface functionalization of AuNPs with mannose, the C2 epimer of glucose, can be a good therapeutic agent for lung cancer treatment and glucose-conjugated AuNPs showed a more angiogenic effect on MDA-MB-231 cells in comparison to mannose-, lactose-, or maltose-coated gold glyconanoparticles. Although further research is needed to fully disclose the observed effect due to the orientation of –OH groups, one explanation could be the change in the composition of protein corona layer as a result of the change in hydrophilicity of the NPs surfaces. While the –OH group on C2 in glucose protrudes down, the one in the mannose pointing up makes itself more available for possible hydrogen bonding with proteins and other molecular species and ions having an affinity for –OH groups.

## Supporting Information Available

Thiolation and purification of carbohydrates, calculation of the concentration of AuNPs suspension, and optimization of the concentration of the modified AuNP suspensions and their characterization (PDF)
