# Supplementary material for: Systematic Investigation of Cellular Response to Hydroxyl Group Orientation Differences on Gold Glyconanoparticles
Source: ACS Omega. 2023 Oct 31;8(45):42921–35. doi: 10.1021/acsomega.3c05920 (PMC10652720; doi:10.1021/acsomega.3c05920)
Supplement: Supplementary file 1 — ao3c05920_si_001.pdf [file ao3c05920_si_001.pdf]

SUPPORTING INFORMATION

Systematic Investigation of Cellular Response to Hydroxyl Group Orientation Differences on Gold Glyconanoparticles

Melike Sarıçam[1], Merve Ercan Ayra[1], Mustafa Culha[1,2,3*]

[1] Department of Genetics and Bioengineering, Yeditepe University, Istanbul 34755, Turkey

[2]Department of Chemistry & Biochemistry, Augusta University, Augusta, Georgia 30912, United States

[3]Sabanci University Nanotechnology Research and Application Center (SUNUM), Istanbul 34956, Turkey

[*]mustafa.culha@sabanciuniv.edu, mculha2@gmail.com.

Thiolation of Carbohydrates

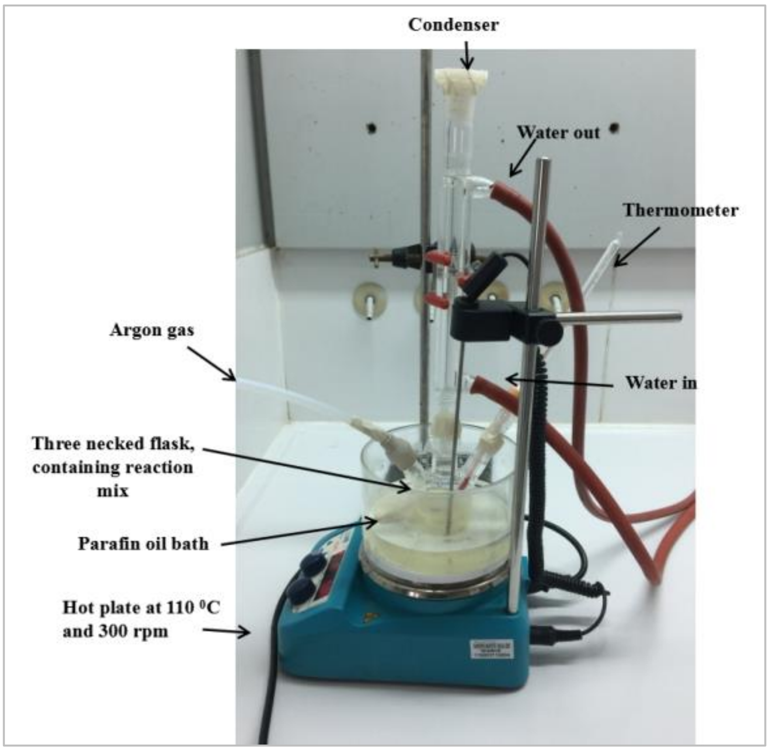

Figure S1. Image of the carbohydrate thiolation set up.

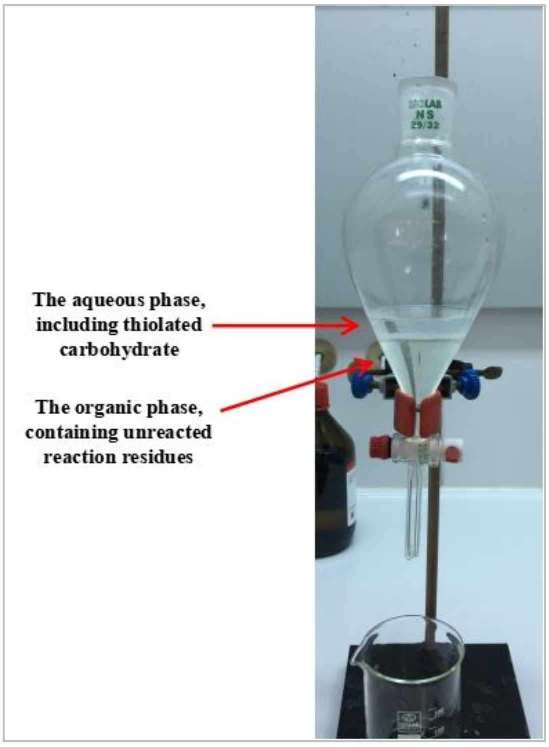

Figure S2. White light image of purification setup of thiolated carbohydrates.

Calculation of AuNP Suspension Concentration

The determination of size and concentration of AuNPs in the suspension are the key points in their applications. In this study, the concentration of synthesized spherical AuNPs in the suspension was determined by Beer-Lambert's Law and compared with theoretical mathematical calculation. Theoretical calculation is based on successive assumptions, which are that the synthesis completely occurs and all AuNPs in the suspension are synthesized in the same size without any standard deviation. On the other hand, in reality it almost impossible to obtain exact monodispersed AuNPs with a unique size in the suspension. Therefore, it would be inaccurate to determine the concentration of AuNPs theoretically. Since SPR properties of synthesized AuNPs can be monitored by UV/Vis spectroscopy, the concentration of AuNPs in suspension can be calculated with low error estimation via Beer-Lambert's Law (Haiss et al., 2007).

To determine the concentration of AuNPs in suspension by Beer-Lambert's Law, the diluted AuNP suspensions in water were prepared as 1:2, 1:4, 1:8 and 1:16 in triplicates. Their absorbance values at the SPR peak (519 nm) were recorded three times by UV/Vis spectroscopy

and the obtained nine measurements for each dilution factors were averaged. The average absorbance values of diluted samples were given in Table S1.

Table S1. Dilution factors of AuNP suspensions and their absorbance values at SPR peak.

Dilution Factor	Absorbance
0.5	1.75
0.25	0.87
0.125	0.44
0.0625	0.23

By using these absorbance values and dilution factors, the calibration curve of synthesized 13 nm AuNP suspension was drawn, as shown in Figure S3.

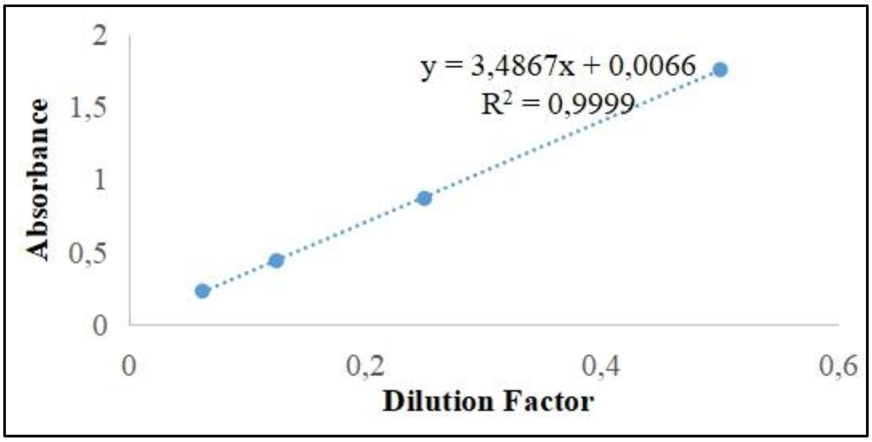

Figure S3. Calibration curve of 13 nm AuNP suspension.

Beer-Lambert's law is referred as:

$$A=C*\varepsilon*l$$

where A is absorbance of NP suspension, C is the concentration of NPs in suspension, ε is extinction coefficient of NPs and l is the path length of cuvette, which was 1 cm. The slope of drawn graph, shown in Figure S4, was calculated as 3.49 and this slope was equal to ε of synthesized AuNPs in suspension from formula ε=A/DF. By referring ε values from literature, ε of around 13 nm sized AuNPs has 10^8 M^{-1} cm^{-1} unit (1). Moreover, A_{spr}/A_{450} ratios discussed by Haiss et. all to determine the concentration of 12 and 14 nm AuNP suspension were 1.56

and 1.61 respectively. The ratio of synthesized 13 nm AuNPs was calculated as 1.57. These complementary results demonstrated that the unit of ε of 13 nm sized AuNPs was 10^8 M^{-1} cm^{-1}. Therefore, ε was assessed as 3.49×10^8 M^{-1} cm^{-1}, as mentioned in Figure S2.

The slope of A vs DF graph = Extinction coefficient

$\varepsilon = 3.49\times10^8\ M^{-1}\ cm^{-1}$

The Concentration of AuNP suspension by using Lambert-Beer's Law:

C=A/ε*DF

Figure S4. Calculation of extinction coefficient of 13 nm AuNP suspension.

By considering the dilution factors, absorbance values at those factors and calculated ε, the concentration of AuNPs in suspension was calculated via Beer-Lambert's Law formula as stated in Table S2. As a result, the concentration of synthesized 13 nm AuNPs suspension was found as **10 nM.**

Table S2. Concentration values of diluted 13 nm AuNP suspensions, which calculated with Beer-Lambert's Law.

Dilution Factor	Absorbance	Concentration C=A/ε*DF
0.5	1.75	1.01E-08
0.25	0.87	9.96E-09
0.125	0.44	1.01E-08
0.0625	0.23	1.07E-08

The total AuNP numbers in 1 ml suspension was calculated by using a formula, referred by Haiss et. Al, as shown in Figure S5. Based on this formula, absorbance value at 450 nm was crucial to designate the AuNP numbers in the suspension. Based on the calculations, it can be concluded that 5.37×10^{12} AuNPs were totally suspended in 1 ml suspension.

The number of AuNPs in 1 ml colloidal suspension:

$$N = \frac{A_{450} \times 10^{14}}{d^2[-0.295 + 1.36\exp(-(\frac{d-96.8}{78.2})^2)]}$$

$$N = 5.37 \times 10^{12} \text{ AuNPs in 1 ml suspension}$$

Figure S5. Determination of AuNP numbers in 1 ml suspension. A_{450} was 1.1026 of 1:10 diluted AuNP suspension and d was equal to 6.5 nm.

The concentration of synthesized AuNPs in suspension was calculated theoretically, as shown mathematically in Figure S6. According to that, the maximum number of 13 nm sized AuNPs in 1 ml colloidal suspension and the maximum concentration of the colloidal suspension can be as 8.7×10^{12} AuNPs and 14.5 nM, respectively.

The weight of Au taken for AuNP synthesis:

$$\left(80\times10^{-3}\right)\mathbf{g}\times0.49=0.0392\ \mathbf{g\ Au}$$

Density of Au = 19.3 g/cm³

The volume of Au taken :

$$\frac{0.0392\ \mathbf{g}}{19.3\ \mathbf{g/cm^3}}=0.002\ \mathbf{cm}^3$$

Average size of a single AuNP: 13.0 nm

Volume of a single AuNP:

$$\frac{4}{3}\times\pi\times\left(6.5\ \mathbf{nm}\right)^3=1{,}150.35\ \mathbf{nm}^3$$

The number of single AuNPs in 200 ml colloidal suspension:

$$\frac{0.002\ \mathbf{cm}^3\times\dfrac{1\ \mathbf{nm}^3}{10^{-21}\ \mathbf{cm}^3}}{1{,}150.35\ \mathbf{nm}^3}=1.74\times10^{15}\ \mathbf{AuNPs}$$

The number of single AuNPs in 1 ml colloidal suspension:

$$\frac{1.74\times10^{15}\ \mathbf{AuNPs}}{200\ \mathbf{ml}}=8.7\times10^{12}\ \mathbf{AuNPs\ in\ 1\ ml}$$

Concentration of AuNP suspension:

$$\frac{1.74\times10^{15}}{6.02\times10^{23}}=2.9\times10^{-9}\ \mathbf{mol}=\mathbf{2.9\ nmol}$$

$$\frac{\mathbf{2.9\ nmol}}{\mathbf{0.2\ L}}=\mathbf{14.5\ nM}$$

Figure S6. Theoretical calculation of the concentration of AuNPs suspension.

In comparison to the experimental and theoretical calculation of AuNP concentration in the suspension, the spherical AuNPs with 13 nm diameter were synthesized by Turkevich method with high monodispersity since the concentration of AuNP suspension (10 nM) and the total number of AuNPs in the suspension (5.37×10^{12}) were close to the theoretical values.

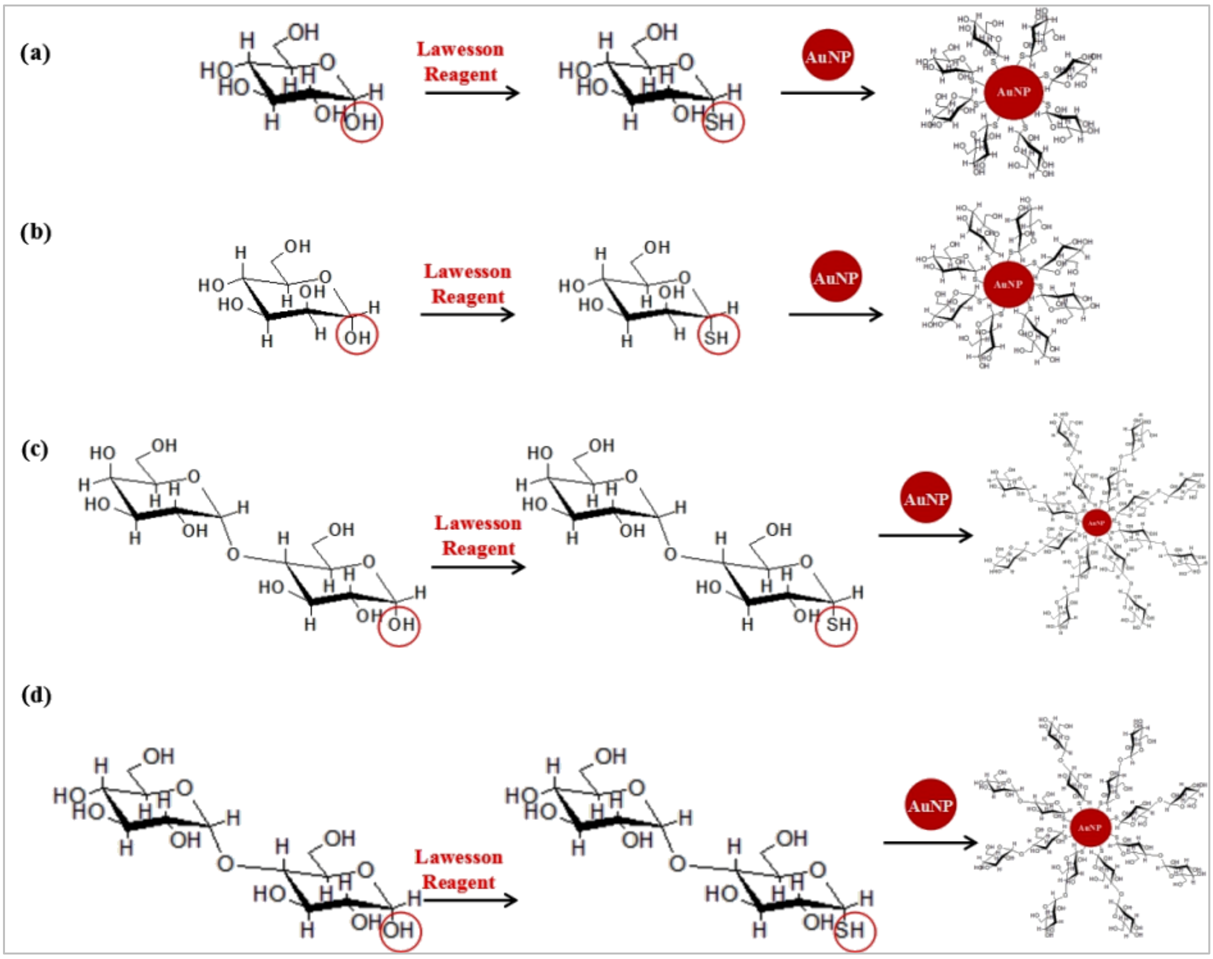

Figure S7. Scheme of AuNP-Carbohydrate conjugation process. **(a)** Glucose, **(b)** Mannose, **(c)** Lactose and **(d)** Maltose.

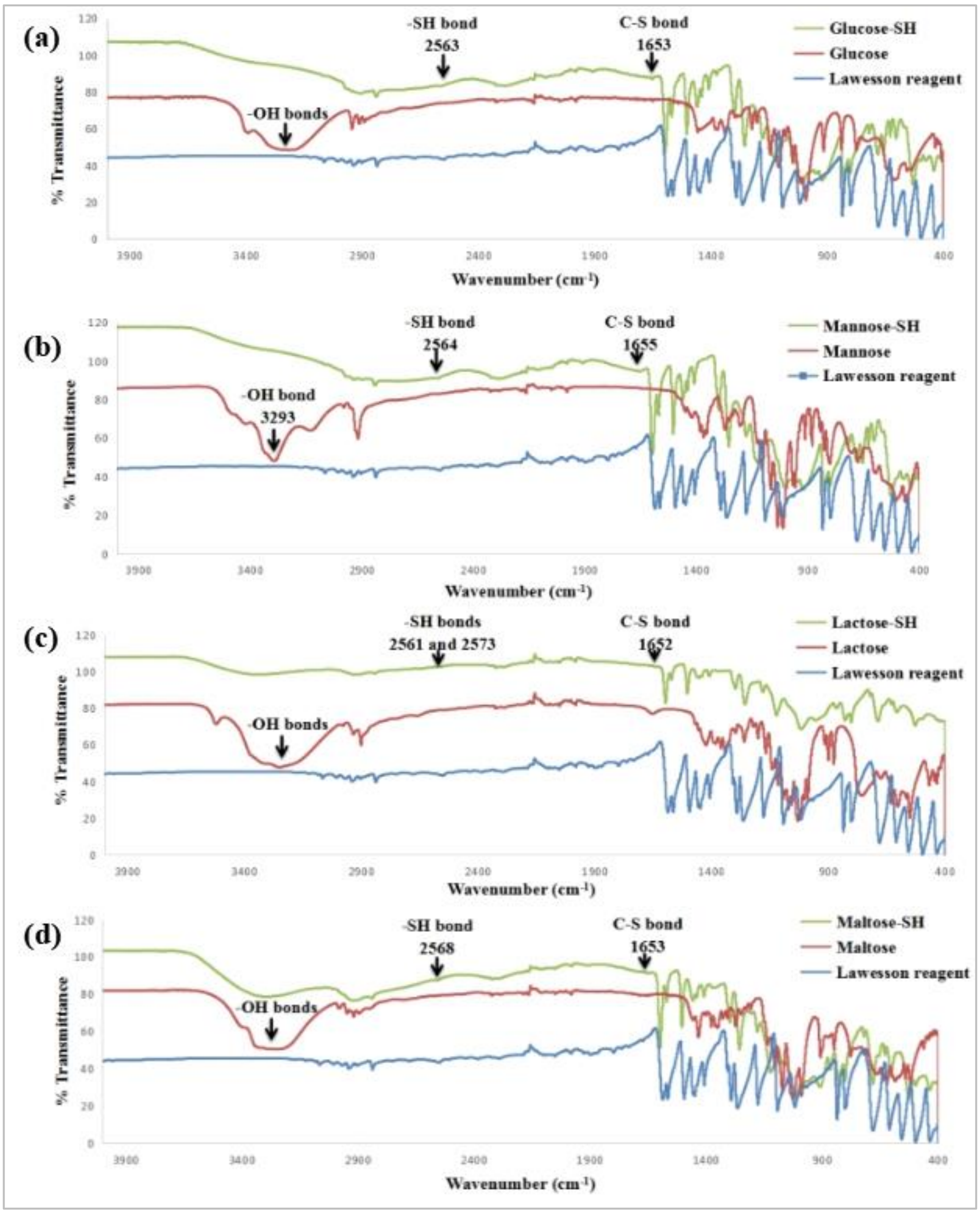

Figure S8. Comparative FTIR spectra of **(a)** Thiolated Glucose, **(b)** Thiolated Mannose, **(c)** Thiolated Lactose and **(d)** Thiolated Maltose.

Optimization of AuNP-Maltose and AuNP-Lactose Conjugates and Their Characterization

The synthesized AuNPs suspension (10 nM, 1ml) were mixed with increasing concentration of 10 mg/ml thiolated Maltose and Lactose solutions (n=3) and incubated for overnight. The white photo of naked AuNPs, AuNP-Maltose and AuNP-Lactose conjugates was given in Figure S9. The color of AuNP conjugate suspensions were darker than the naked AuNPs suspension and

no AuNP aggregation was observed after the conjugation indicating the stability of the suspension.

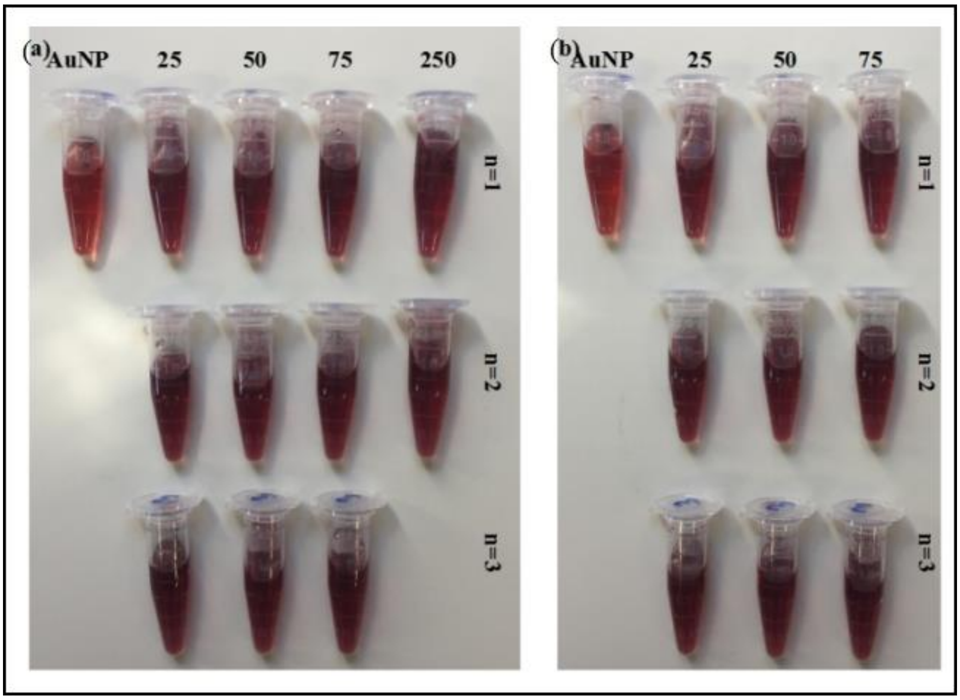

Figure S9. While light images of suspensions of (a) naked AuNP and AuNP-Maltose conjugates, and (b) naked AuNP and AuNP-Lactose conjugates. "n" indicates replicate number.

The naked AuNPs and AuNP-Maltose and AuNP-Lactose conjugates were characterized using UV/Vis spectroscopy, DLS and 1% Agarose gel electrophoresis as seen in Figure S10. As a conclusion, the 5-6 nm redshift on UV/Vis spectra, bigger hydrodynamic sizes, more negative zeta potentials and dense bands on the agarose gel indicated that the surfaces of AuNPs in suspension (10 nM, 1 ml) were conjugated with Maltose and Lactose solution successfully, and 75 µl of 10 mg/ml of Maltose and Lactose were enough to equally coat all AuNPs in 1 ml suspension.

a)

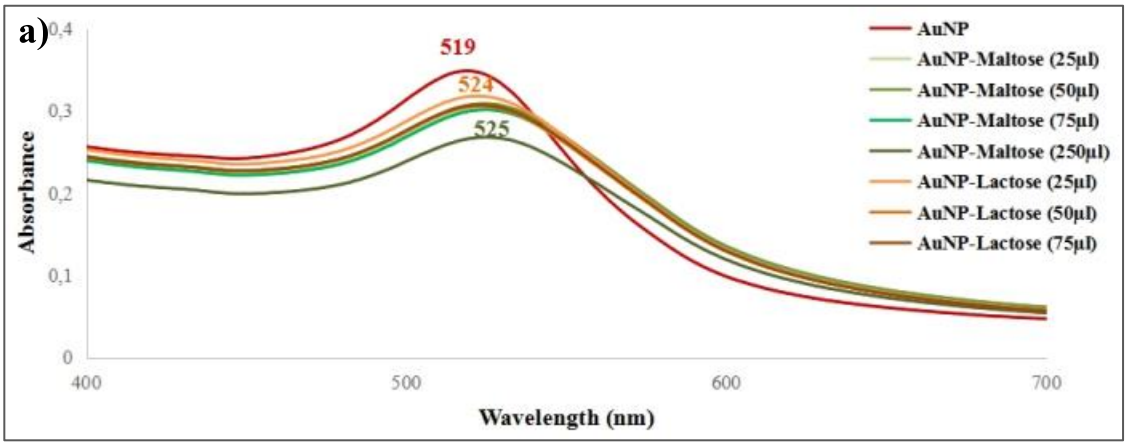

b)

Sample	Size (nm)	Zeta potential (mV)
AuNPs	12.2 ± 0.3	-13.3 ± 0.2
AuNP-Maltose(25µl)	13.4 ± 0.5	-21.4 ± 2.6
AuNP-Maltose (50µl)	13.3 ± 0.7	-16.1 ± 1.0
AuNP-Maltose (75µl)	13.9 ± 0.6	-26.9 ± 6.6
AuNP-Maltose (250µl)	13.8 ± 0.4	-23.7 ± 2.4
AuNP-Lactose (25µl)	13.3 ± 0.3	-20.4 ± 4.9
AuNP-Lactose (50µl)	13.0 ± 0.7	-26.2 ± 3.4
AuNP-Lactose (75µl)	13.2 ± 0.2	-22.1 ± 5.7

c)

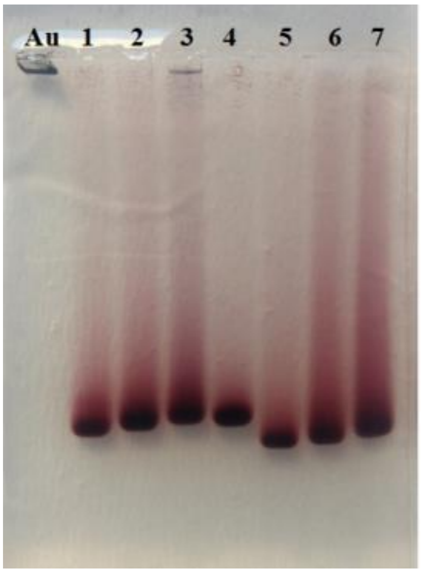

Au: AuNPs
1: AuNP-Maltose (25 µl)
2: AuNP-Maltose (50 µl)
3: AuNP-Maltose (75 µl)
4: AuNP-Maltose (250 µl)
5: AuNP-Maltose (25 µl)
6: AuNP-Maltose (50 µl)
7: AuNP-Maltose (75 µl)

Figure S10. Characterization of naked AuNPs, AuNP-Maltose and AuNP-Lactose conjugate suspensions. a) UV/Vis spectra, b) Average hydrodynamic sizes and zeta potentials and c) White light image of agarose gel.

Optimization of AuNP-Glucose and AuNP-Mannose Conjugates and Their Characterization

The spherical AuNPs of 13 nm diameter size (10 nM, 1ml) were coated with increasing concentrations of 10 mg/ml Glucose and Mannose, as same as AuNP-Lactose and AuNP-Maltose conjugates. The photo of naked AuNPs and AuNP-Carbohydrate conjugates was given in Figure S11. The color of AuNP conjugate suspensions were darker than the naked AuNPs suspension and no AuNP aggregation was observed in the reaction tubes after conjugation indicating the stability of the suspension.

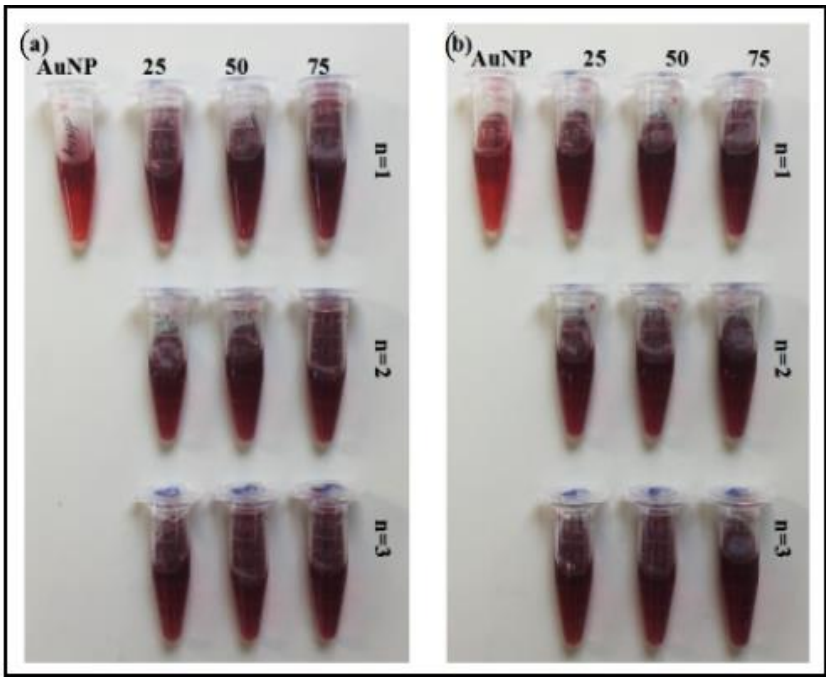

Figure S11. While light images of suspensions of (a) naked AuNP and AuNP-Glucose conjugates, and (b) naked AuNP and AuNP-Mannose conjugates. “n” indicates replicate number.

The naked AuNPs and AuNP-Glucose and AuNP-Mannose conjugates were characterized by UV/Vis spectroscopy, DLS and 1% Agarose gel elctrophoresis as seen in Figure S12. The 4-6 nm redshift on UV/Vis spectra, larger hydrodynamic sizes and more negative zeta potentials were implied the conjugation of AuNPs with mono saccharides. On the other hand, AuNP-Glucose and AuNP-Mannose conjugates run through the gel as smear, which meant that the concentration of mono saccharides (25, 50 and 75 µl of 10 mg/ml) was not enough to coat all AuNPs surfaces in 1 ml suspension.

a)

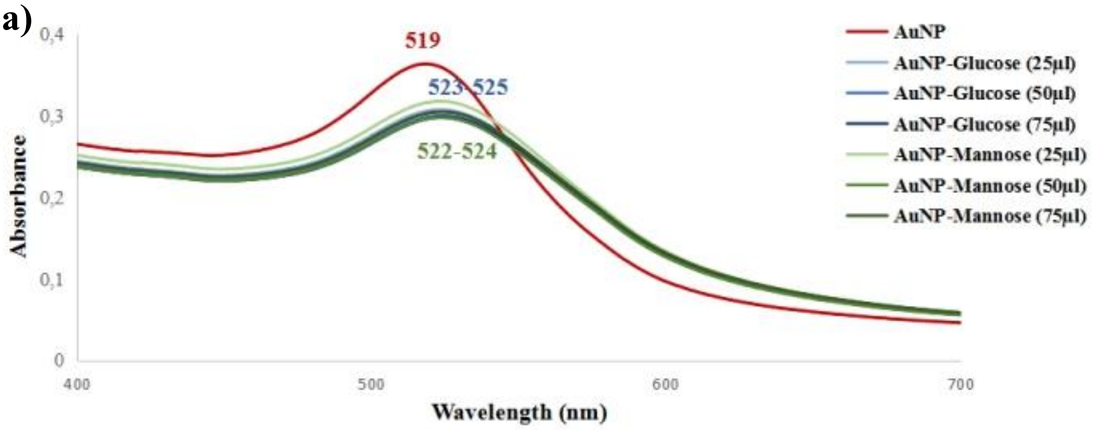

b)

Sample	Size (nm)	Zeta potential (mV)
AuNPs	11.7 ± 0.4	-20.0 ± 1.3
AuNP-Glucose (25µl)	12.9 ± 0.5	-31.3 ± 8.0
AuNP-Glucose (50µl)	13.0 ± 0.4	-45.3 ± 4.6
AuNP-Glucose (75µl)	13.0 ± 0.8	-34.0 ± 9.5
AuNP-Mannose (25µl)	12.6 ± 0.5	-42.3 ± 6.0
AuNP-Mannose (50µl)	12.5 ± 0.5	-36.0 ± 11.0
AuNP-Mannose (75µl)	12.1 ± 0.8	-26.6 ± 6.4

c)

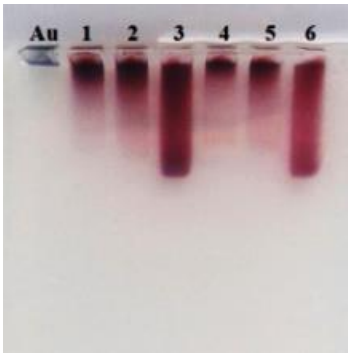

Au: AuNPs
1: AuNP-Glucose (25 µl)
2: AuNP-Glucose (50 µl)
3: AuNP-Glucose (75 µl)
4: AuNP-Mannose (25 µl)
5: AuNP-Mannose (50 µl)
6: AuNP-Mannose (75 µl)

Figure S12. Characterization of naked AuNPs and AuNP conjugates functionalized with 25, 50 and 75 µl of 10 mg/ml Glucose and Mannose solutions. a) UV/Vis spectra, b) Average hydrodynamic sizes and zeta potentials and c) White light image of agarose gel.

In order to functionalization of AuNPs with higher concentrations of thiolated Glucose and Mannose solutions, the spherical AuNPs of 13 nm diameter size (10 nM, 1ml) were conjugated with 100, 150 and 200 µl of 10 mg/ml thiolated Glucose and Mannose solutions (n=3). They were shaken for overnight. The suspensions of naked AuNPs and AuNP-Glucose and AuNP-Mannose conjugates were seen in Figure S13. The color of AuNP conjugate suspensions were darker than the naked AuNPs suspension and no AuNP aggregation was observed after the functionalization indicating the stability of the suspension.

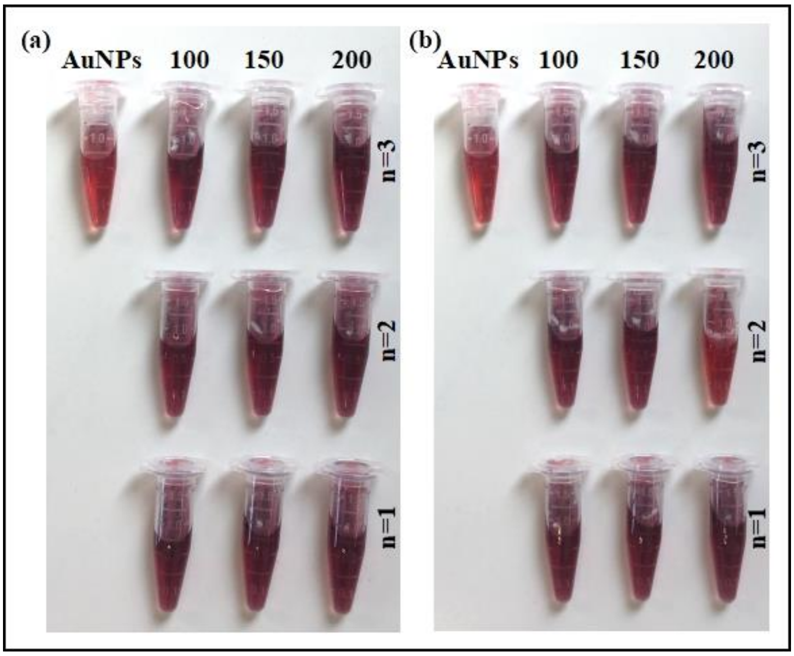

Figure S13. White light images of suspensions of (a) naked AuNP and AuNP-Glucose conjugates, and (b) naked AuNP and AuNP-Mannose conjugates. “n” indicates replicate number.

The naked AuNPs, AuNP-Glucose and AuNP-Mannose conjugates were characterized by UV/Vis spectroscopy, DLS and 1% Agarose electrophoresis, as seen Figure S14. The 5 nm redshift on UV/Vis spectra, the grown hydrodynamic sizes, the more negative zeta potentials and the dense red bands run on the agarose gel (especially 150-200 µl of 10 mg/ml mono saccharide solution added conjugates) showed that the surfaces of AuNPs in 1 ml suspension should be functionalized with 150 µl of 10 mg/ml thiolated Glucose and Mannose solution to obtain dense coating.

a)

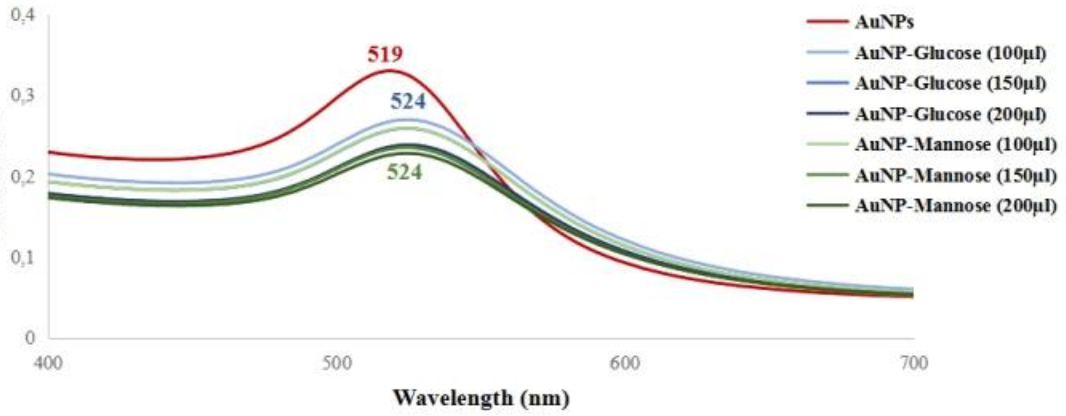

b)

Sample	Size (nm)	Zeta potential (mV)
AuNPs	11.9 ± 0.3	-6.1 ± 1.7
AuNP-Glucose (100µl)	12.2 ± 0.8	-10.8 ± 3.1
AuNP-Glucose (150µl)	12.6 ± 0.5	-32.2 ± 8.3
AuNP-Glucose (200µl)	12.7 ± 0.5	-26.2 ± 1.7
AuNP-Mannose (100µl)	12.2 ± 0.9	-9.4 ± 0.4
AuNP-Mannose (150µl)	12.3 ± 0.7	-13.8 ± 1.7
AuNP-Mannose (200µl)	12.6 ± 0.7	-22.7± 1.8

c)

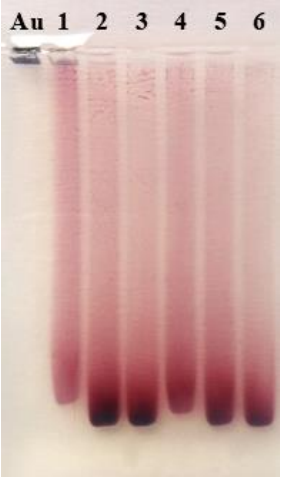

Au: AuNPs
1: AuNP-Glucose (100 µl)
2: AuNP-Glucose (150 µl)
3: AuNP-Glucose (200 µl)
4: AuNP-Mannose (100 µl)
5: AuNP-Mannose (150 µl)
6: AuNP-Mannose (200 µl)

Figure S14. Characterization of naked AuNPs and AuNP conjugates functionalized with 100, 150 and 200 µl of 10 mg/ml Glucose and Mannose solutions. a) UV/Vis spectra, b) Average hydrodynamic sizes and zeta potentials and c) White light image of agarose gel.

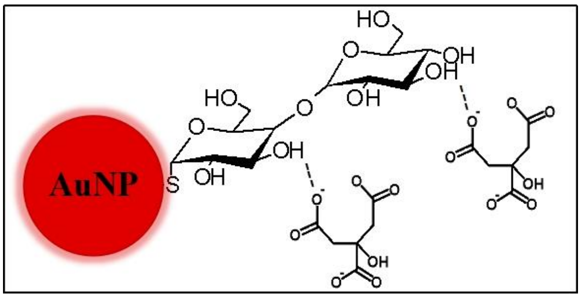

Figure S15. Intermolecular interaction between the free citrate ions and -OH groups of carbohydrates on AuNP surfaces.

References

Haiss, W., Thanh, N. T. K., Aveyard, J., & Fernig, D. G. (2007). Determination of size and concentration of gold nanoparticles from UV− Vis spectra. *Analytical Chemistry*, *79*(11), 4215–4221.
